# Laparoscopic versus open gastrectomy for nonmetastatic T4a gastric cancer: a meta-analysis of reconstructed individual participant data from propensity score-matched studies

**DOI:** 10.1186/s12957-024-03422-5

**Published:** 2024-05-29

**Authors:** Huayang Pang, Menghua Yan, Zhou Zhao, Lihui Chen, Xiufeng Chen, Zhixiong Chen, Hao Sun, Yunyun Zhang

**Affiliations:** 1 Department of Gastrointestinal Cancer Center, Chongqing University Cancer Hospital, Chongqing, 400030 China; 2 Chongqing Key Laboratory of Translational Research for Cancer Metastasis and Individualized Treatment, Chongqing University Cancer Hospital, Chongqing, 400030 China

**Keywords:** Gastric cancer, T4a, Laparoscopic gastrectomy, Open gastrectomy, Reconstructed survival curves, Meta-analysis

## Abstract

**Background:**

​The applicability of laparoscopy to nonmetastatic T4a patients with gastric cancer remains unclear due to the lack of high-quality evidence. The purpose of this study was to compare the survival rates of laparoscopic gastrectomy (LG) versus open gastrectomy (OG) for these patients through a meta-analysis of reconstructed individual participant data from propensity score-matched studies.

**Methods:**

PubMed, Embase, Web of Science, Cochrane library and CNKI were examined for relevant studies without language restrictions through July 25, 2023. Individual participant data on overall survival (OS) and disease-free survival (DFS) were extracted from the published Kaplan-Meier survival curves. One-stage and two-stage meta-analyses were performed. In addition, data regarding surgical outcomes and recurrence patterns were also collected, which were meta-analyzed using traditional aggregated data.

**Results:**

Six studies comprising 1860 patients were included for analysis. In the one-stage meta-analyses, the results demonstrated that LG was associated with a significantly better DFS (Random-effects model: *P* = 0.027; Restricted mean survival time [RMST] up to 5 years: *P* = 0.033) and a comparable OS (Random-effects model: *P* = 0.135; RMST up to 5 years: *P* = 0.053) than OG for T4a gastric cancer patients. Two-stage meta-analyses resulted in similar results, with a 13% reduced hazard of cancer-related death (*P* = 0.04) and 10% reduced hazard of overall mortality (*P* = 0.11) in the LG group. For secondary outcomes, the pooled results showed an association of LG with less estimated blood loss, faster postoperative recovery and more retrieved lymph nodes.

**Conclusion:**

Laparoscopic surgery for patients with nonmetastatic T4a disease is associated with a potential survival benefit and improved surgical outcomes.

**Supplementary Information:**

The online version contains supplementary material available at 10.1186/s12957-024-03422-5.

## Background

Gastric cancer (GC) is currently one of the most common malignancies worldwide [1]. ​Although significant advances have been achieved with adjuvant therapies, surgery with curative intent remains the most important treatment strategy for patients with gastric cancer [2]. In the era of minimally invasive surgery, several large-scale randomized controlled trials (RCTs) have demonstrated that laparoscopic gastrectomy (LG) has comparable oncological efficacy with improved surgical outcomes compared with open gastrectomy (OG) in the treatment of advanced gastric cancer (AGC) [3–5]. Based on this evidence, the present NCCN and ESMO guidelines both recommend laparoscopic approach as an alternative for advanced (T2-T4a) nonmetastatic gastric cancer patients [6, 7].

Nevertheless, current studies of LG for AGC have mainly focused on T2-T3 patients, with relatively fewer T4a patients included. According to the AJCC staging system, T4a is defined as tumor invasion of serosa [8], which is characterized by large tumor size, high risk of recurrence/metastasis, and poor prognosis [9]. For such patients, LG faces higher surgical challenges and may increase the risk of peritoneal seeding. Until now, there have been no prospective studies on LG for T4a gastric cancer patients, and only one RCT [10] have focused on this issue by performing subgroup analysis. Therefore, the effectiveness of LG for T4a gastric cancer patients is still a topic of concern for clinical practice, especially regarding long-term survival outcomes.

To provide an evidence-based basis for future update to these guidelines, we performed an individual participant data (IPD) meta-analysis of survival outcomes from propensity-score matched (PSM) studies, which compared LG versus OG for nonmetastatic T4a gastric cancer patients. Pooled analysis using IPD is regarded as the gold standard in evidence synthesis, and is widely accepted as the most reliable approach in current practice [11]. In addition, to overcome the selection and confounding bias inherent in most observational studies, we limited studies to those that performed PSM analysis, because numerous statistical studies have demonstrated that PSM studies are empirically equivalent to RCTs in obtaining unbiased estimates of the so-called “average treatment effect” [12, 13].

## Methods

This meta-analysis was performed in adherence to the requirements from the PRISMA 2020 statement [14]. This study has been registered at PROSPERO (registration number: CRD42023420723).

### Search strategy

PubMed, Embase, Web of Science, Cochrane library and China National Knowledge Infrastructure were comprehensively examined for relevant studies without language restrictions through July 25, 2023. The detailed search strategy of each database was shown in Supplementary file item 1. In addition, Google Scholar, the references of included articles, and related reviews were manually searched for potential gray literature.

### Inclusion and exclusion criteria

The inclusion criteria were determined according to Population, Intervention, Comparison, Outcome, and Study design (PICOS) [15] approach: (1) P: patients diagnosed with clinical (c), surgical (s) or pathological (p) T4a gastric cancer; (2) I: LG; (3) C: OG; (4) O: survival outcomes with Kaplan-Meier curves reported; (5) S: observational studies based on PSM analysis, or RCTs. Exclusion criteria: (1) studies without matching, or using other matching methods of confounder control, such as stratification, and inverse probability of treatment weighting; (2) studies in the form of reviews, conferences, case reports, letters, and expert opinions; (3) duplicated studies; (4) original data were not available from the relevant authors.

### Data extraction and outcome of interest

Two investigators independently extracted the data into a predefined EXCEL table and cross-checked all the results. Any disagreements were resolved by consensus with a third reviewer. The following data were extracted: (1) study characteristics; (2) patient baseline parameters; (3) perioperative outcomes; (4) survival outcomes and recurrence patterns.

The primary outcomes were to compare survival outcomes between the LG and OG groups. The survival outcomes included overall survival (OS), disease-free survival (DFS) and recurrence-free survival (RFS). Of note, since DFS and RFS share the similar endpoints, they were analyzed together as one outcome, DFS [16, 17]. The secondary outcomes were to compare the surgical outcomes (operative time, estimated blood loss, number of retrieved lymph nodes, time to first liquid intake, time to first flatus, postoperative hospital stay, overall morbidity and major morbidity) and recurrence patterns. Morbidities were defined according to the Clavien–Dindo classification, and major complications were defined as grade III or higher [18].

### Assessment of the quality of evidence

Two reviewers assessed the quality of included studies using the Newcastle-Ottawa Scale [19] (NOS). Briefly, the NOS evaluates 8 items in 3 key domains: selection, comparability and outcome. The score ranges from 0 to 9. The quality of each study was categorized into 3 levels via the total points obtained: low (< 4 points), moderate (between 4 and 6 points), and high (≥ 7 points).

### Statistical analysis

#### Reconstruction of time-to-event outcomes

Patient-level survival data were extracted from published survival curves according to the methods reported by Guyot et al. [20]. Briefly, Kaplan-Meier curves from included studies were digitized using the Digitizelt software. Then, the survival information was algorithmically restored based on the numerical solution of the inverted Kaplan–Meier product-limit equations, and any departures from monotonicity were corrected using a pool-adjacent-violators algorithm. In addition, summary statistics from individual studies such as survival percentages, hazard ratios (HRs), number-at-risk tables or total number of events were used to improve the calibration of the time-to-events.

#### Survival analysis

The Kaplan-Meier method was performed to calculate survival outcomes. One-stage survival meta-analyses were conducted using Cox proportional hazards models and restricted mean survival time (RMST) [21]. We modeled between-study heterogeneity using two approaches. Firstly, the primary analysis was based on the shared frailty model, which incorporates a random-effects model in which individual participants within each study are assumed to be similarly failure-prone as other individuals belonging to that study [22]. Across studies, frailties are gamma distributed and affect the hazard function in a latent, multiplicative manner [22]. Secondly, we used stratified Cox models to adjust for inter-study heterogeneity by allowing patients from a given study to assume a baseline hazard unique to that study [23]. The Grambsch-Therneau tests for nonzero slope with a plotted scaled Schoenfeld residuals were applied to identify violations of the proportionality assumption of Cox regression models [24]. In addition, differences in survival outcomes between the LG and OG groups were also assessed via RMST, which can provide a robust estimation of survival at different cut-off time points in the presence of proportionality violation [25]. Finally, as a sensitivity analysis, a conventional two-stage meta-analysis of aggregated HRs (based on the reconstructed individual patient dataset) using Inverse-Variance weighted random-effects was performed [11].

#### Meta-analysis of aggregated patient data

The mean differences (MDs) and risk ratios (RRs) with corresponding 95% confidence intervals (CIs) were used as the effect sizes for continuous effects and dichotomous effects, respectively. For studies that reported median with range or interquartile range, the McGrath et al. [26] method was used to estimate the mean with standard deviation. Heterogeneity of effect sizes among included studies was assessed using I^2^ statistic. Random-effects models were applied to balance inherent clinical heterogeneity across included studies [27]. Publication bias was assessed via Egger’s test for each outcome, and trim and fill analysis was employed when there was a significant publication bias. A two-tailed *P* value < 0.05 was considered statistically significant. All analyses were performed using Review Manager Software, version 5.3 (Cochrane, London, UK), Stata, version 12.0 (Statacorp, College Station, TX) and R software, version 4.2.1 (R Group for Statistical Computing).

## Results

### Study characteristics

**Fig. 1:**
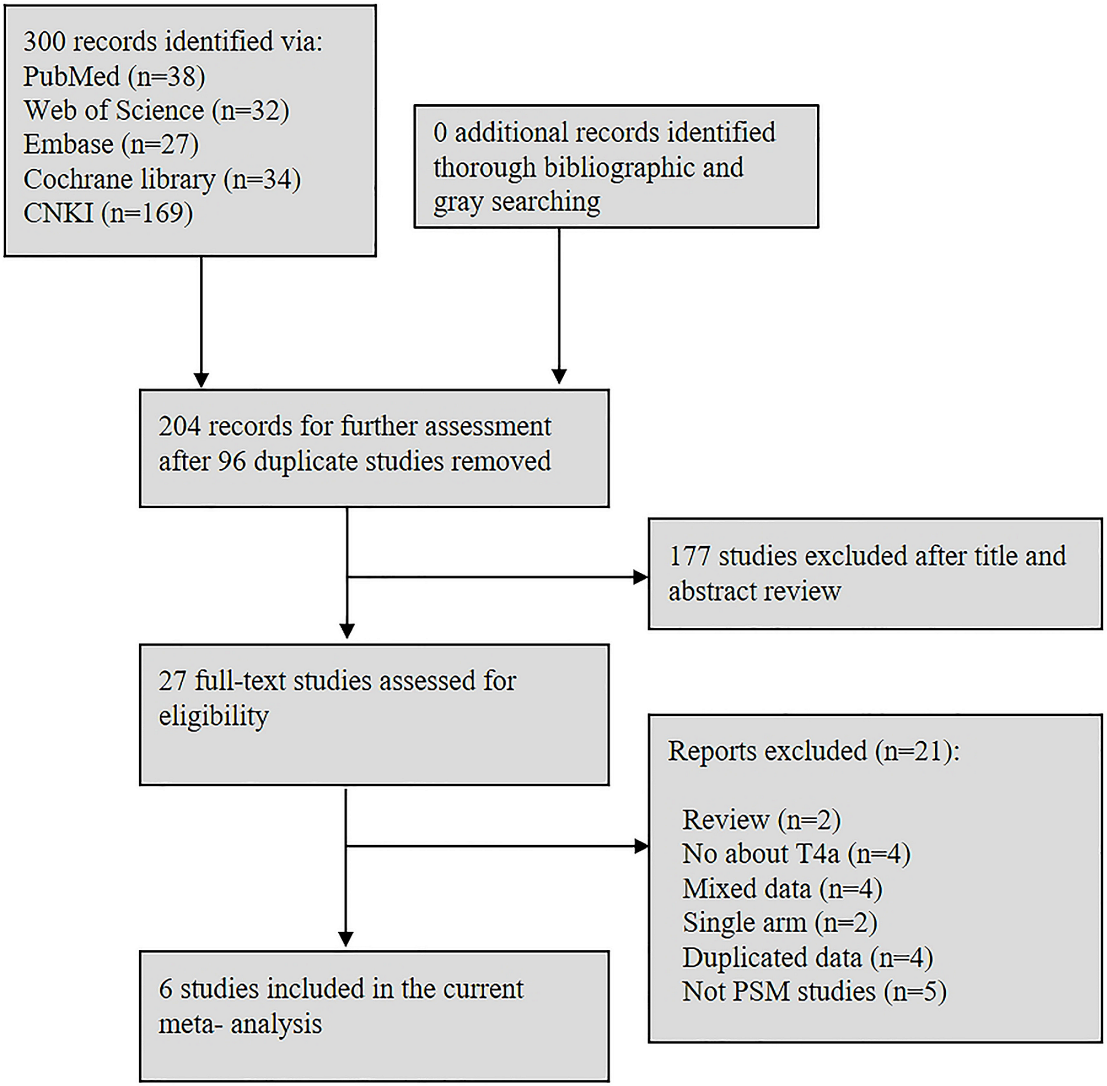
Flow chart of study selection

The search strategy yielded a total of 300 potentially relevant studies. After title de-duplication and abstract assessment, 27 full-texts were reviewed, of which 21 references were excluded for various reasons (Fig. 1). Of note, 4 studies [10, 28–30] were excluded due to overlapping data, and 5 studies were excluded because PSM analysis was not performed [31–35]. In all, 6 retrospective PSM studies [36–41] and no RCT, comprising 1860 patients (930 in the LG group and 930 in the OG group) were included. These studies were published between 2019 and 2023, and carried out in China, Korea, Japan and Vietnam. Among these studies, 5 of them included only pT4a patients, whereas inclusion was based on sT4a (91.2% pT4a patients) disease in one study. For the outcomes, all of the included studies reported OS, and 5 studies reported DFS/RFS. Additionally, all PSM studies were judged by two reviewers independently using NOS checklist and had an NOS score of 7 or 8 stars, indicating that they were of sufficient quality (Table 1 and Supplementary file item 2).

**Table 1:** Study characteristics of included studies

Reference	Country	Study design	Study period	Inclusion and exclusion criteria	Variables matched	Sample size (LG: OG)	Median follow-up, months	Quality score
Jeong,2022	Korea	R; S	2005–2017	Inclusion: pT4aN0-3M0 gastric cancer; curative resection. Exclusion: positive margin	Age, sex, BMI, comorbidity, surgical procedure, level of lymph node dissection, and pN stage	248 (124:124)	38	7
Kuwabara,2023	Japan	R; S	2002–2016	Inclusion: pT4aN0-3M0 gastric adenocarcinoma; curative resection. Exclusion: remnant gastric cancer, with other malignant diseases, emergency operation.	Age, sex, preoperative treatment, comorbidity, surgical procedure, and pN stage.	90(45:45)	60	8
Li,2019	China	R; S	2009–2015	Inclusion: pT4aN0-3M0 gastric adenocarcinoma, age between 18 and 70 years. Exclusion: emergency surgery, previous chemotherapy or surgery for gastric cancer.	Age, sex, BMI, surgical procedure, ASA grade, tumor size, tumor differentiation, pN stage and pTNM stage	404(202:202)	57	8
Long,2021	China	R; S	2004–2014	Inclusion: pT4aN0-3M0 gastric adenocarcinoma, curative resection, age between 18 and 80 years. Exclusion: with other malignant diseases, neo-adjuvant therapy, emergency operation.	Age, sex, BMI, ASA grade, tumor size, pN stage, surgical procedure, and tumor differentiation	668(334:334)	97	8
Long,2022	Vietnam	R; S	2013–2020	Inclusion: sT4aN0–3M0 gastric adenocarcinoma. Exclusion: intraoperatively detected bulky lymph nodes, inadequate lymphadenectomy (D0/1/1+), R2 resection, ASA grade ≥ 4, with other malignant diseases, previous chemotherapy or surgery for gastric cancer, emergency operation.	Age, sex, BMI, ASA grade, comorbidities, gastric outlet obstruction, tumor differentiation, tumor size, adjuvant chemotherapy, and type of anastomosis	294(147:147)	LG:31.6; OG:50.3	8
Pang,2021	China	R; S	2006–2016	Inclusion: pT4aN0-3M0 gastric adenocarcinoma, curative resection. Exclusion: remnant gastric cancer, with other malignant diseases, preoperative oncologic treatment, conversion to open surgery, proximal gastrectomy.	Age, sex, surgical procedure, tumor size, macroscopic type, tumor differentiation, pTNM stage, and adjuvant chemotherapy	156(78:78)	96.4	8

LG: laparoscopic gastrectomy; OG: open gastrectomy; R: retrospective; S: single center

As shown in Supplementary file item 3, no significant differences were observed in baseline characteristics between the LG and OG groups (All P values > 0.05).

### Primary outcomes

**Fig. 2:**
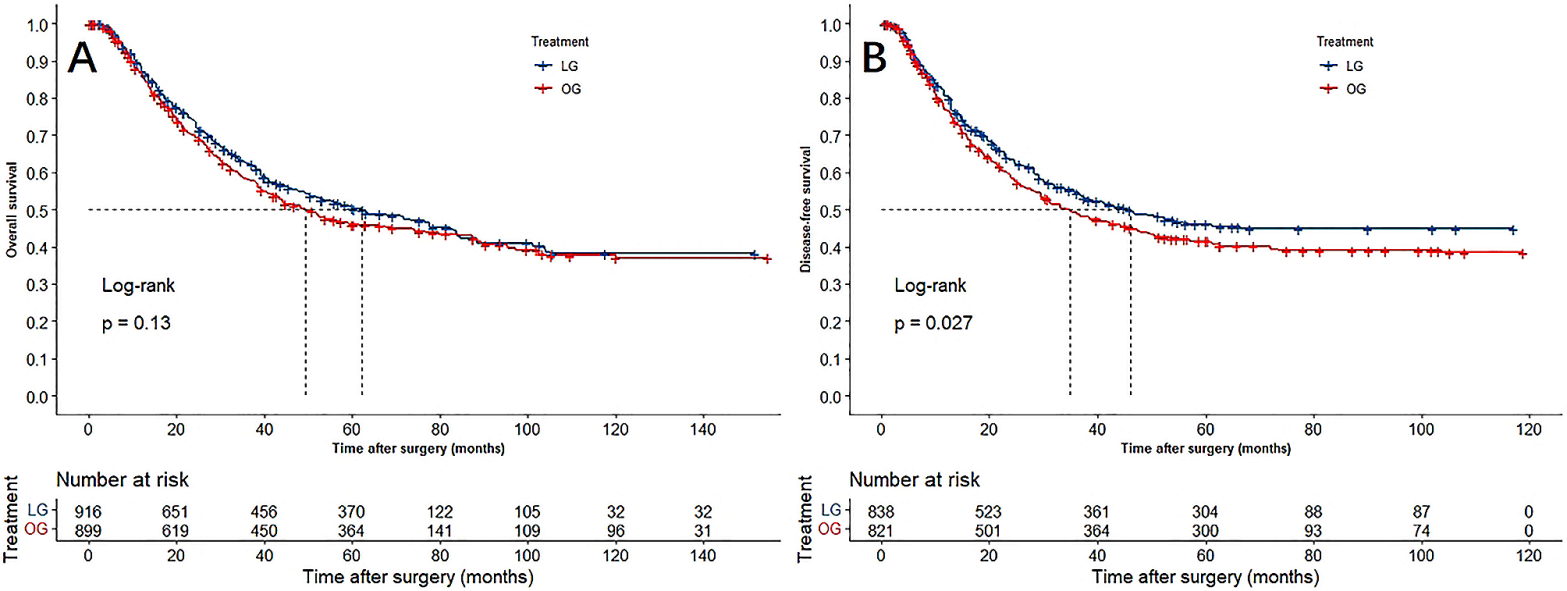
Kaplan-Meier curves for LG versus OG in patients with T4a gastric cancer. A: overall survival; B: disease-free survival

The reconstructed survival curves and visually side-by-side comparison with the original curve were shown in Supplementary file item 4. The reconstructed and published curves in each study were nearly identical, and the discrepancies in the risk tables were negligible. The OS and DFS curves of the combined population were shown in Fig. 2. The 1-year OS rate in the LG group was 88.4%, 3-year OS rate, 62.5%, and 5-year OS rate, 50.4%; in the OG group, the 1-year OS rate was 86.7%, 3-year OS rate, 58.3%, and 5-year OS rate, 46.0%. For DFS, the 1-year, 3-year and 5-year DFS rate in the LG group was 80.4%, 54.5% and 46.1%, and 76.9%, 49.1%, and 41.5% in the OG group.

In the Cox-based shared-frailty model, the combined analysis of OS yielded a non-significant HR of 0.907 (95%CI: 0.797–1.031; *P* = 0.135). While compared with OG, LG was associated with significantly better DFS with an HR of 0.861 (95%CI: 0.755–0.983; *P* = 0.027). Analyses via the stratified Cox model to help adjust for inter-study heterogeneity yielded similar estimates (Table 2).

**Table 2:** Primary and sensitivity analyses of survival outcomes using reconstructed survival information

	Overall survival				Disease-free survival		
	Relative effect (95%CI)	P value	Test of non-PH		Relative effect (95%CI)	P value	Test of non-PH
**Semiparametric models**							
Random-effects HR (Shared frailty)	0.907 (0.797–1.031)	0.135	0.624		0.861 (0.755–0.983)	0.027	0.883
Stratified Cox HR	0.905 (0.794–1.032)	0.134	0.597		0.859 (0.751–0.982)	0.026	0.881
**Nonparametric models**							
RMST difference (up to 1 year)	0.100 (-0.056-0.256)	0.210			0.159 (0.072–0.178)	0.178	
RMST ratio (up to 1 year)	1.009 (0.995–1.022)	0.210			1.014 (0.993–1.036)	0.178	
RMST difference (up to 3 years)	0.894 (-0.101-1.888)	0.078			1.260 (0.069–2.450)	0.038	
RMST ratio (up to 3 years)	1.032 (0.996–1.068)	0.078			1.049 (1.003–1.099)	0.038	
RMST difference (up to 5 years)	1.935 (-0.028-3.898)	0.053			2.429 (0.202–4.657)	0.033	
RMST ratio (up to 5 years)	1.048 (0.999–1.099)	0.053			1.067 (1.005–1.134)	0.033	

The RMST analysis was performed to assess the differences in survival time between the LG and OG groups. Compared to OG, the mean OS time at 1-year follow-up was 0.100 month (*P* = 0.210) in favor of LG, and this difference increased to 0.894 (*P* = 0.078) at 3-year and 1.935 (*P* = 0.053) at 5-year. As for DFS, the mean time at 1-year follow-up was 0.159 month (*P* = 0.178) in favor of LG, and this difference increased to 1.260 (*P* = 0.038) at 3-year and 2.429 (*P* = 0.033) at 5-year (Table 2).

**Fig. 3:**
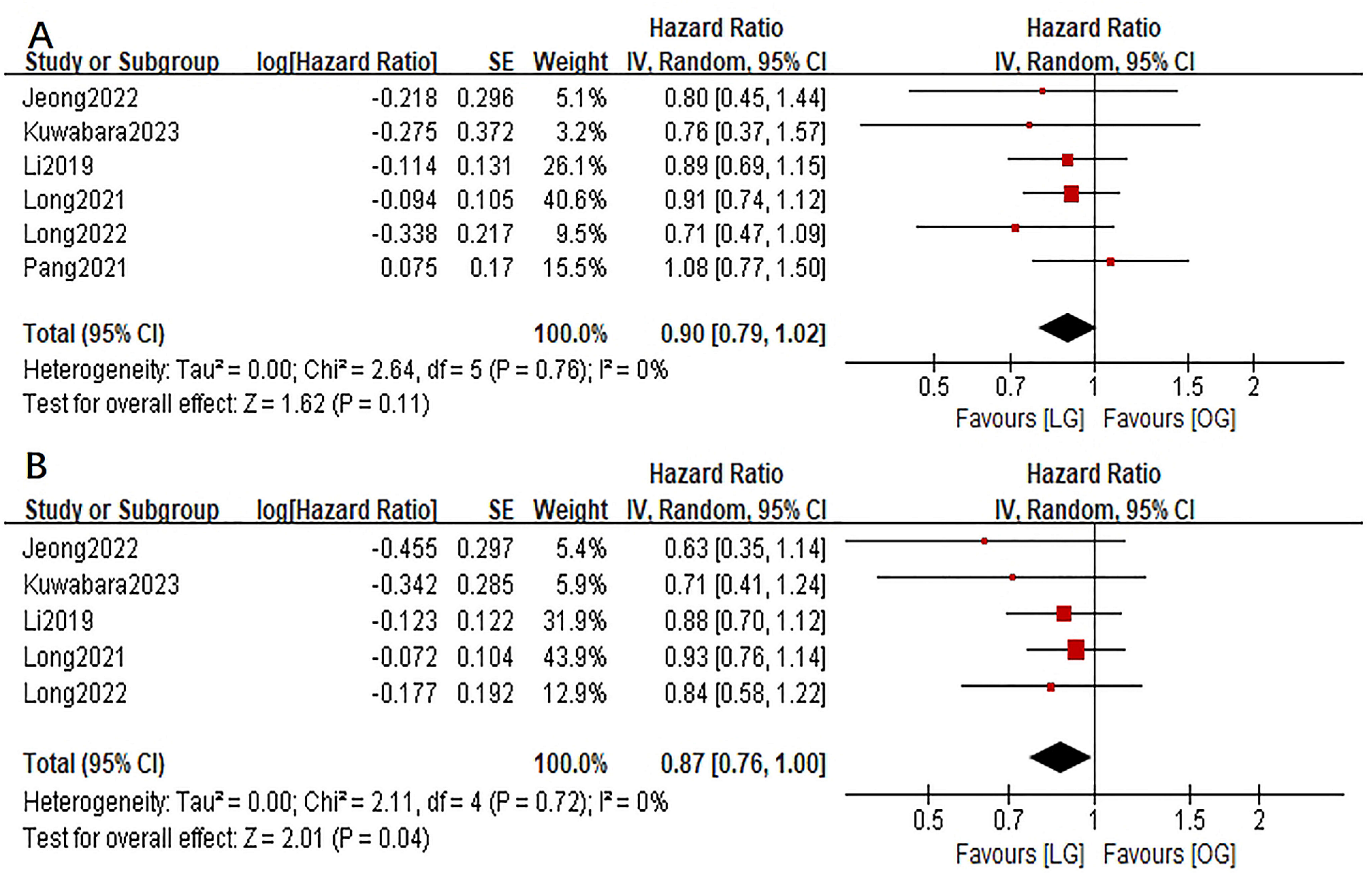
Forest plots assessing survival outcomes between the LG and OG groups in patients with T4a gastric cancer. A: overall survival; B: disease-free survival

In the two-stage meta-analyses, a pooled HR of 0.90 (95%CI:0.79–1.02; *P* = 0.11) for OS and a pooled HR of 0.87 (95%CI:0.76-1.00; *P* = 0.04) for DFS were observed, which was almost the same as the HRs in the one-stage analyses (Fig. 3). Both the analyses of OS and DFS had no obvious heterogeneities (both I^2^ = 0%).

### Secondary outcomes

#### Surgical outcomes

**Fig. 4:**
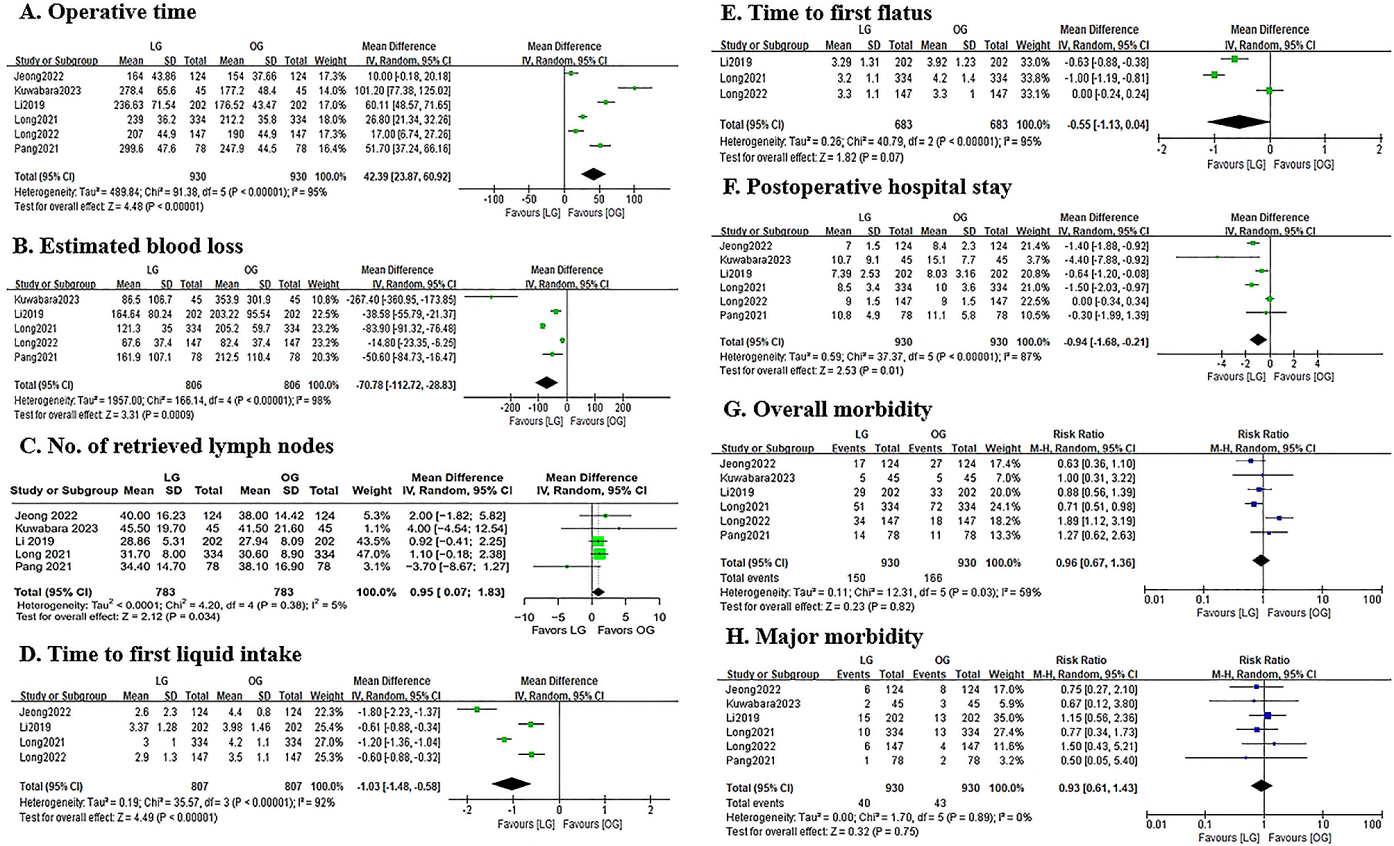
Forest plots assessing surgical outcomes including (A) operative time, (B) estimated blood loss, (C) no. of retrieved lymph nodes, (D) time to first liquid intake, (E) time to first flatus, (F) postoperative hospital stay, (G) overall morbidity and (H) major morbidity between the LG and OG groups

As shown in Fig. 4, the LG group was associated with a longer operative time (MD = 42.39; 95%CI:23.87–60.92; *P* < 0.0001; I^2^ = 95%), less estimated blood loss (MD=-70.78; 95%CI: -112.72 to -28.83; *P* = 0.0009; I^2^ = 98%) and more harvested lymph node (MD = 0.95; 95%CI:0.07–1.83; *P* = 0.034; I^2^ = 5%). The time to first liquid diet (MD=-1.03; 95%CI: -1.48 to -0.58; *P* < 0.0001; I^2^ = 92%) and postoperative hospital stay (MD=-0.94; 95%CI: -1.68 to -0.21; *P* = 0.01; I^2^ = 87%) were shorter in the LG group than those in the OG group. In addition, the LG group had a marginally shorter time to first flatus (MD=-0.55; 95%CI: -1.13-0.04; *P* = 0.07; I^2^ = 95%). No significant differences were observed in the overall (RR = 0.96; 95%CI: 0.67–1.36; *P* = 0.82; I^2^ = 82%) and major complications (RR = 0.93; 95%CI: 0.61–1.43; *P* = 0.75; I^2^ = 0%) between the LG and OG groups.

### Recurrence patterns

**Fig. 5:**
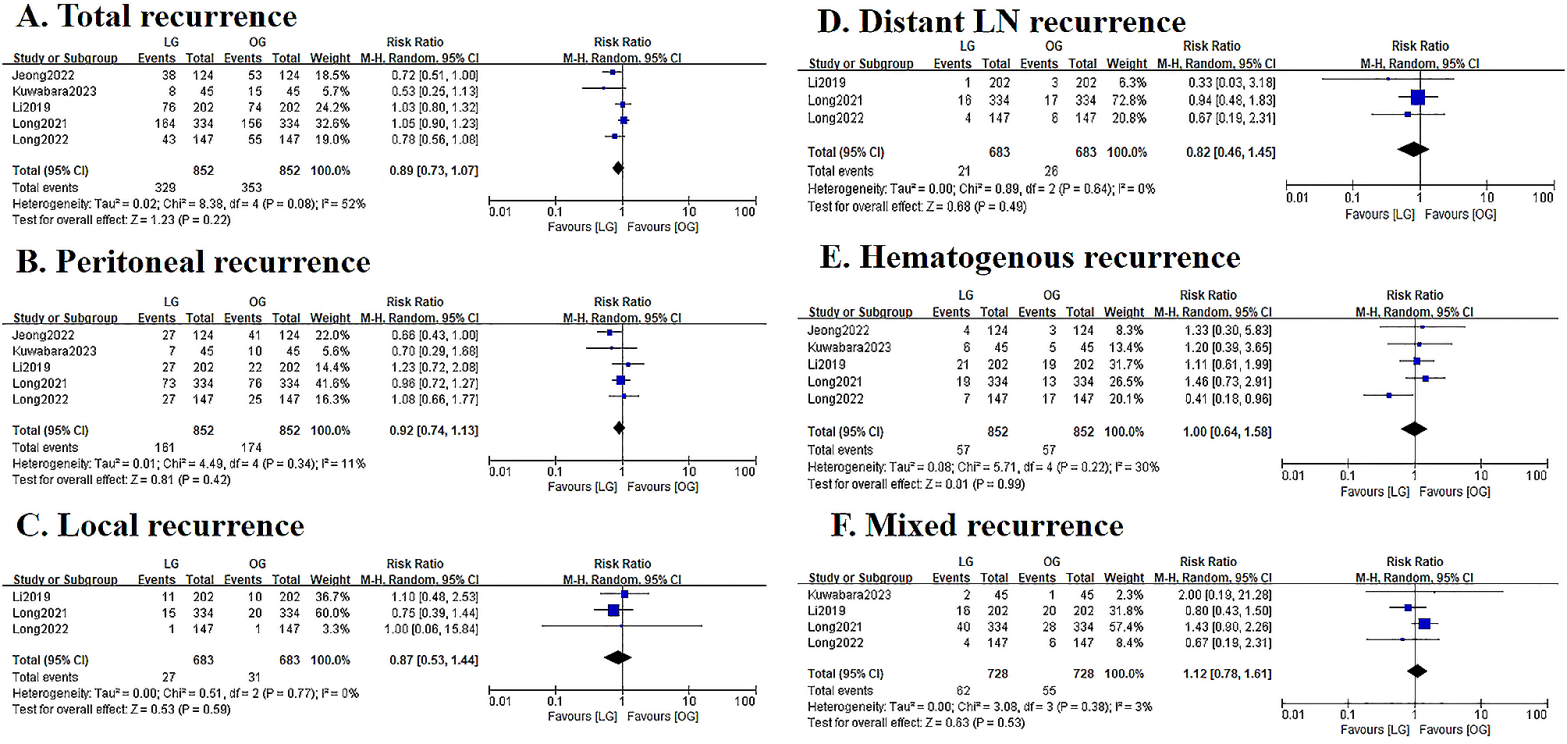
Forest plots assessing recurrence patterns including (A) Total recurrence, (B) Peritoneal recurrence, (C) Local recurrence, (D) Distant LN recurrence, (E) Hematogenous recurrence, and (F) Mixed recurrence between the LG and OG groups

As shown in Figs. 5 and 38.62% in the LG group and 41.43% in the OG group developed recurrence, and the recurrence rate was not significant different (RR = 0.89; 95%CI: 0.73–1.07; *P* = 0.22; I^2^ = 52%). Peritoneal seeding was the most common site of recurrence in both groups (18.90% vs. 20.42%), while with no significant difference (RR = 0.92; 95%CI: 0.74–1.13; *P* = 0.42; I^2^ = 11%). Besides, there were no significant differences between the two groups in terms of locoregional recurrence (RR = 0.87; 95%CI: 0.53–1.44; *P* = 0.59; I^2^ = 0%), distant lymph node metastasis (RR = 0.82; 95%CI: 0.46–1.45; *P* = 0.49; I^2^ = 0%), hematogenous metastasis (RR = 1.00; 95%CI: 0.64–1.58; *P* = 0.99; I^2^ = 30%) as well as mixed recurrence (RR = 1.12; 95%CI: 0.78–1.61; *P* = 0.53; I^2^ = 3%).

### Publication bias

For both primary and secondary outcomes, the Egger’s tests were conducted to assess the potential publication bias. As shown in Supplementary file item 5, all of the pooled outcomes except DFS (*P* = 0.007), showed no significant risk of publication bias. Trim-and fill analysis was further performed to test the reliability of the pooled outcome of DFS, showing that 0 unpublished studies needed to be supplemented, indicating an unchanged pooled result.

## Discussion

In this meta-analysis, a total of 6 PSM studies comprising 1860 nonmetastatic T4a gastric cancer patients were included. The analysis demonstrated that LG was associated with a significantly better DFS and a comparable OS than OG for patients with T4a gastric cancer. In addition, the pooled results showed improved benefits in perioperative parameters, with an association of LG with less estimated blood loss, faster postoperative recovery and more retrieved lymph nodes.

​Although the safety and feasibility of LG in advanced gastric cancer has been extensively demonstrated in both Eastern and Western countries, the existing data in the literature raise concerns about the advantages in T4a patients. The most important factor in determining the feasibility of a new surgical approach for oncologic surgery is long-term survival, especially OS. This IPD meta-analysis of PSM studies was therefore conducted to summarize the best available evidence using rigorous statistical methodologies, and suggested a potential survival benefit in favor of laparoscopic versus open surgery for these patients. These findings are promising and provocative as it is reassuring to gastrointestinal surgeons who routinely perform LG. From a conservative insight, these results can be interpreted to indicate that laparoscopic surgery is at least not inferior to the standard open approach.

Consistent comparable OS and DFS of laparoscopic and open gastrectomy for nonmetastatic T4a patients have been reported across included studies. The potential survival benefit in favor of LG in our study may seem surprising, but we can find some clues from previous studies. From the perspective of survival curves, with the exception of the studies by Jeong et al. [36] and Pang et al. [41], other studies demonstrated a clear separation in overall survival curves between the LG and OG groups. Regarding DFS, all studies except the one by Long et al. [39] also showed a distinct separation in survival curves between the two groups over extended periods of follow-up. The same trend was observed in terms of reported survival rates, for example, at 5 years after surgery, Long et al. [40] reported an OS rate of 69% in the LG group and 60% in the OG group. Kuwabara and colleagues [37] reported a 5-year DFS rate showing a 12.2% advantage of LG compared with OG (51.3% vs. 39.1%). Nevertheless, none of the differences were statistically significant, which may be caused by insufficient statistical power due to the limited sample size of the individual studies. By conducting a combined analysis of survival data with a larger sample size and employing multiple robust statistical methods, we definitively demonstrated that the LG group exhibited superior DFS compared to the OG group. For OS, although no statistically significant difference was observed in the present study, we believe that, at least on the current IPD survival curve, there may be a survival benefit for LG with a longer follow-up. In addition, we also compared the baseline characteristics between the LG and OG groups based on the available data. The pooled results showed that after PSM, there were no statistical differences between the baseline characteristics of the two groups. A good baseline balance will be more conducive to proving the reliability of our conclusions.

We surmise that the potential survival benefit conferred by LG is not solely a statistical artifact, but also reflects the existence of some underlying clinical and biological mechanisms. Several reasons may explain the potential survival benefit associated with laparoscopic approach. First, less blood loss in the LG group may decrease the likelihood of tumor spillage and hematogenous spread [42]. ​A multicenter cohort study has confirmed that intraoperative bleeding was an independent prognostic factor of disease recurrence in locally advanced gastric cancer patients, and the effect became more significant in stage III patients [43]. Second, the improved postoperative recovery after LG could allow patients to receive subsequent adjuvant treatment earlier [44]. A large body of evidence has demonstrated that the delay in postoperative chemotherapy was associated with adverse survival outcomes in patients with gastric cancer [45]. Third, more examined lymph nodes are associated with accurate TNM staging and prolonged survival in gastric cancer patients [46]. ​The removal of an adequate number of lymph nodes is an important indicator assessing the feasibility of laparoscopy in this group of patients and is also a concern for many surgeons. However, in the present study, LG was associated with a higher number of harvested lymph nodes, which could be a contributing factor to the improved survival outcomes. Finally, benefiting from the minimally invasive nature of laparoscopy (e.g., meticulous manipulation and small incisions), laparoscopic surgery did not seem to increase the risk of recurrence (including peritoneal dissemination) in this subset of gastric cancer patients. In addition, compared to the open approach, minimally invasive surgery is beneficial in reducing surgical stress, which has been found to suppress the body’s anti-cancer immune surveillance [47].

### Strengths and limitations

​The present meta-analysis has several strengths. We have included only high-quality PSM studies that can effectively overcome the selection and confounding biases inherent in most observational studies. We have used the most appropriate method to extract data from these studies (i.e., the use of IPD). The extraction of individual participant time-to-event data from published Kaplan-Meier curves allows us to generate more robust results than traditional aggregated data meta-analysis. Additionally, the IPD survival analyses were further validated by two-stage meta-analyses, which showed low heterogeneities (I^2^ = 0% for both OS and DFS). Moreover, we have performed publication bias tests for both primary and secondary outcomes, suggesting good stability and reliability.

There are also some limitations in this meta-analysis. First, the IPD analysis provided only patient-level survival data, and was not able to provide other covariates such as age, BMI and tumor location. ​Due to the relative difficulty of laparoscopic approach in older patients, those with high BMI, or those undergoing total gastrectomy, surgeons have more concerns about the oncological outcomes of LG in those patients. Nonetheless, insufficient study-level relevant data did not allow us to perform such subgroup analyses. Second, all included studies were retrospective, prospective studies and RCTs are still lacking. Third, the included studies were all from Asian countries and Western experience was not reported. Therefore, large-scale multicenter RCTs are still warranted to further investigate the applicability of LG on patients with T4a gastric cancer.

## Conclusion

In the current meta-analysis of PSM studies, potential survival benefits and superior surgical outcomes were found for LG compared to OG for patients with nonmetastatic T4a gastric cancer. These meaningful findings for the laparoscopic approach are encouraging and support the routine use of LG for nonmetastatic T4a gastric cancer patients in experienced centers.

### Electronic supplementary material

Below is the link to the electronic supplementary material.

Supplementary Material 1

Supplementary Material 2

Supplementary Material 3

Supplementary Material 4

Supplementary Material 5

Supplementary Material 6

## Data Availability

The data that support the findings of this study are owned by the participating cohort studies. Data are not publicly available but may be shared upon reasonable request at each cohort depending on cohort‐specific regulations.

## Declarations

### Research Registration Unique Identifying Number (UIN)

(1) Name of the registry: PROSPERO database; (2) Registration ID: CRD42023420723; (3) Hyperlink: https://www.crd.york.ac.uk/prospero/display_record.php?RecordID=420723.
