# Supplementary material for: Laparoscopic versus open gastrectomy for nonmetastatic T4a gastric cancer: a meta-analysis of reconstructed individual participant data from propensity score-matched studies
Source: World J Surg Oncol. 2024 May 29;22:143. doi: 10.1186/s12957-024-03422-5 (PMC11134691; doi:10.1186/s12957-024-03422-5)
Supplement: Supplementary file 2 — Supplementary Material 2 [file 12957_2024_3422_MOESM2_ESM.doc]

Supplementary Item 2. Newcastle-Ottawa Quality Assessment Scale for included studies.

| **Study** | **Selection** | | | | **Comparability** | **Outcome** | | | **Quality score** |
| --- | --- | --- | --- | --- | --- | --- | --- | --- | --- |
|  | Representativeness of the exposed cohort | Selection of the non-exposed cohort | Ascertainment of exposure | Demonstration that outcome of interest was not present at start of study | Comparability of cohorts on the basis of the design or analysis controlled for confounders | Assessment of outcome | Was follow-up long enough for outcomes to occur | Adequacy of follow-up of cohorts | Total score |
| Jeong,2022 | 1 | 1 | 1 | 0 | 1 | 1 | 1 | 1 | 7 |
| Kuwabara,2023 | 1 | 1 | 1 | 0 | 2 | 1 | 1 | 1 | 8 |
| Li,2019 | 1 | 1 | 1 | 0 | 2 | 1 | 1 | 1 | 8 |
| Long,2021 | 1 | 1 | 1 | 0 | 2 | 1 | 1 | 1 | 8 |
| Long,2022 | 1 | 1 | 1 | 0 | 2 | 1 | 1 | 1 | 8 |
| Pang,2021 | 1 | 1 | 1 | 0 | 2 | 1 | 1 | 1 | 8 |
