# Supplementary material for: Laparoscopic versus open gastrectomy for nonmetastatic T4a gastric cancer: a meta-analysis of reconstructed individual participant data from propensity score-matched studies
Source: World J Surg Oncol. 2024 May 29;22:143. doi: 10.1186/s12957-024-03422-5 (PMC11134691; doi:10.1186/s12957-024-03422-5)
Supplement: Supplementary file 3 — Supplementary Material 3 [file 12957_2024_3422_MOESM3_ESM.docx]

**A. Age**


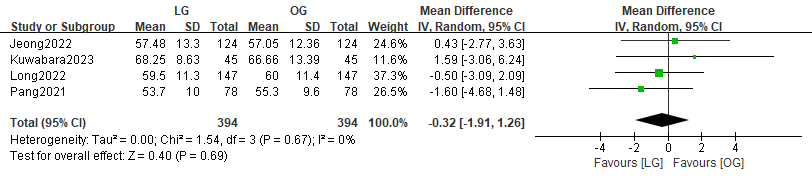


**B. Sex**

**
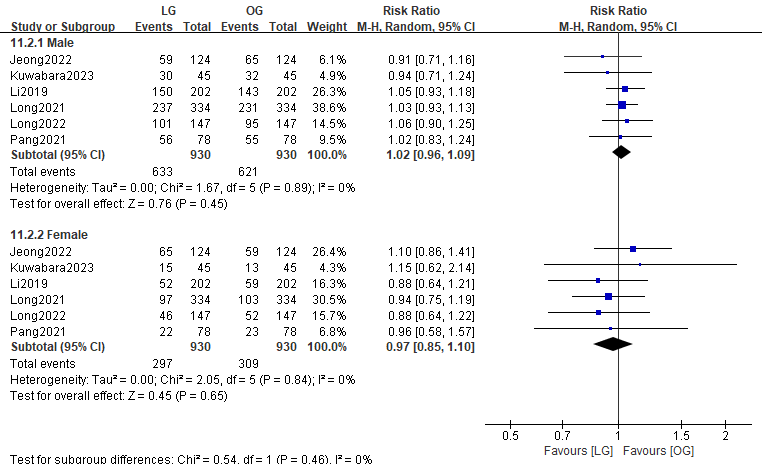
**

**C. BMI**


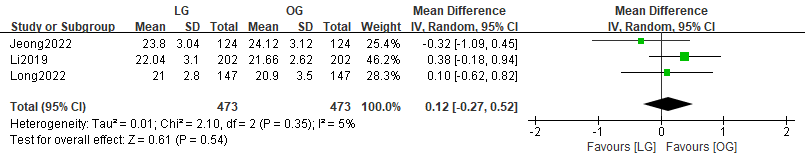


**D. Comorbidities**


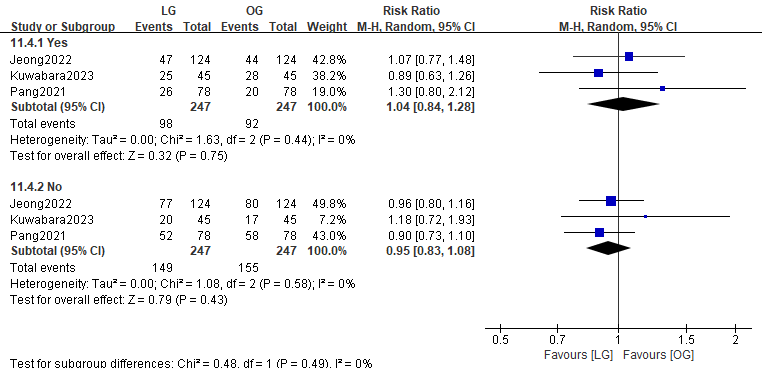


**E. Gastrectomy extent**


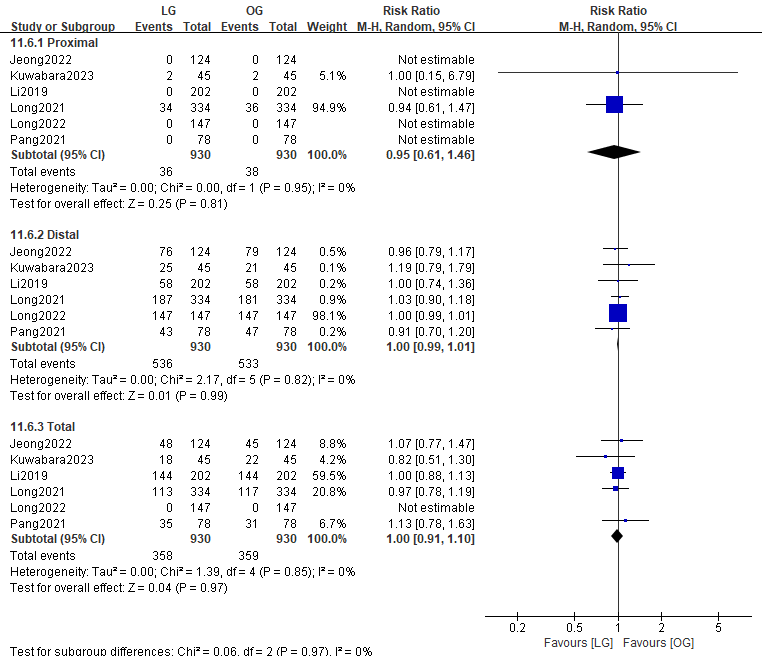


**F. Tumor size**


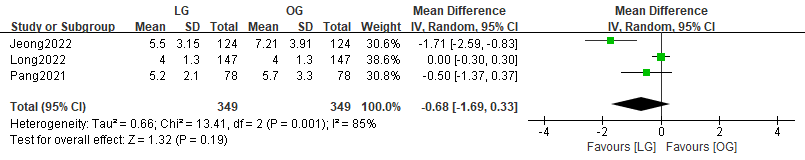


**G. Tumor differentiation**


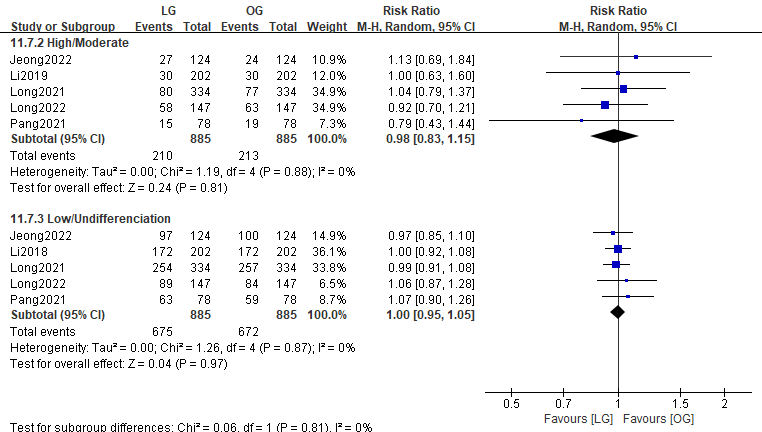


**H. Lymph node involvement**


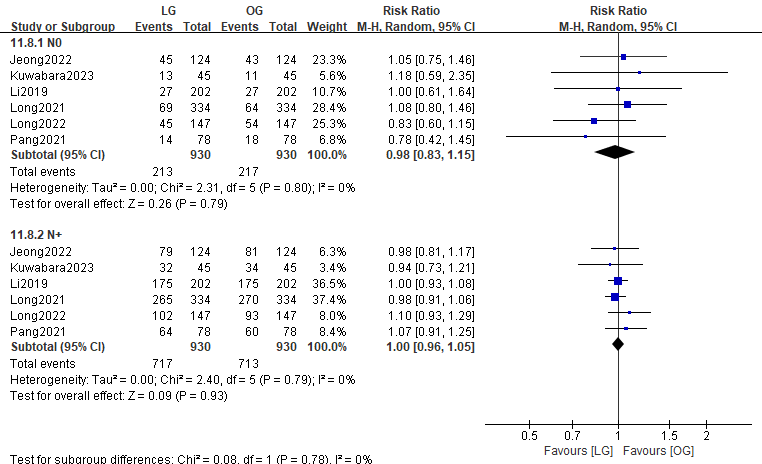


Supplementary file item 3. Forest plots of patient baseline characteristics between the LG and OG groups.
