# Supplementary material for: Laparoscopic versus open gastrectomy for nonmetastatic T4a gastric cancer: a meta-analysis of reconstructed individual participant data from propensity score-matched studies
Source: World J Surg Oncol. 2024 May 29;22:143. doi: 10.1186/s12957-024-03422-5 (PMC11134691; doi:10.1186/s12957-024-03422-5)
Supplement: Supplementary file 4 — Supplementary Material 4 [file 12957_2024_3422_MOESM4_ESM.docx]

Supplementary file Item 1. Search strategies of specific database.

| Database | Search strategy | Search result |
| --- | --- | --- |
| PubMed | 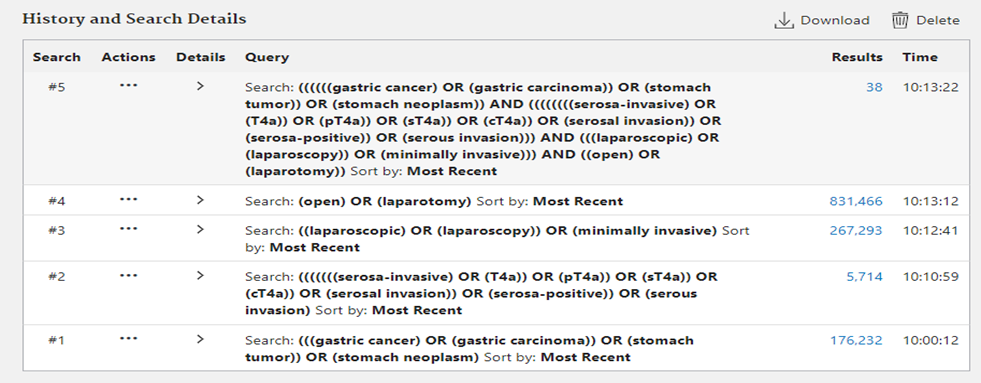 | 38 |
|  | 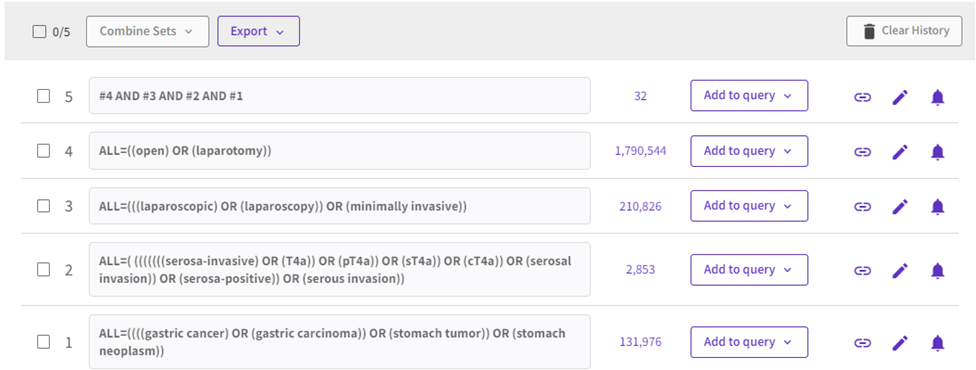 | 32 |
| Embase | 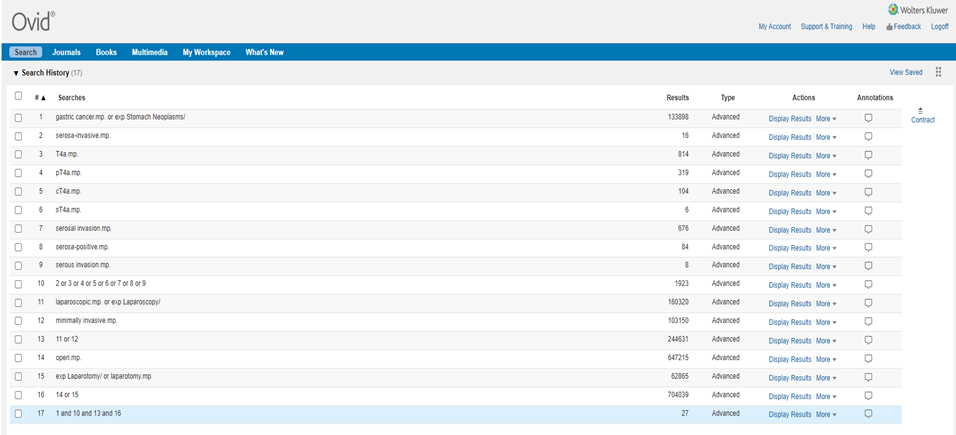 | 27 |
| Cochrane library | 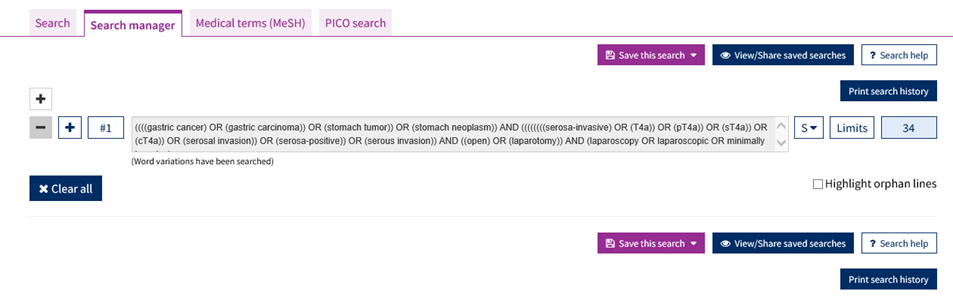 | 34 |
| CNKI | 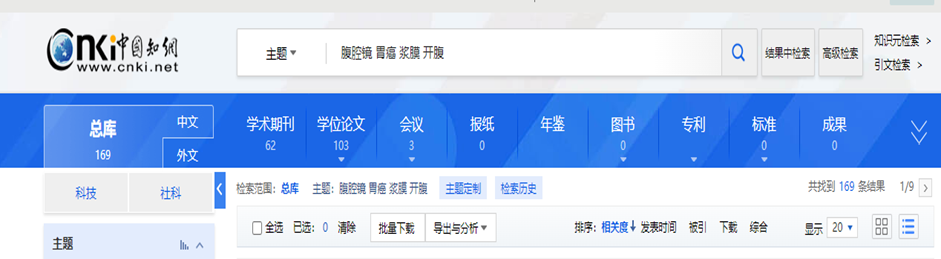 | 169 |
