# Supplementary material for: Laparoscopic versus open gastrectomy for nonmetastatic T4a gastric cancer: a meta-analysis of reconstructed individual participant data from propensity score-matched studies
Source: World J Surg Oncol. 2024 May 29;22:143. doi: 10.1186/s12957-024-03422-5 (PMC11134691; doi:10.1186/s12957-024-03422-5)
Supplement: Supplementary file 5 — Supplementary Material 5 [file 12957_2024_3422_MOESM5_ESM.docx]

Supplementary file item 4. Original and reconstructed survival curves from the included studies.

| References | Original | Reconstructed |
| --- | --- | --- |
| Jeong, 2022  (OS) | 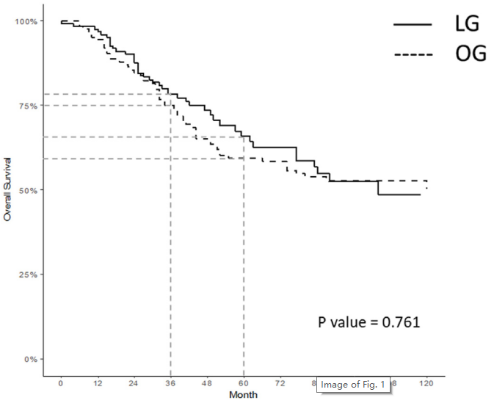 | 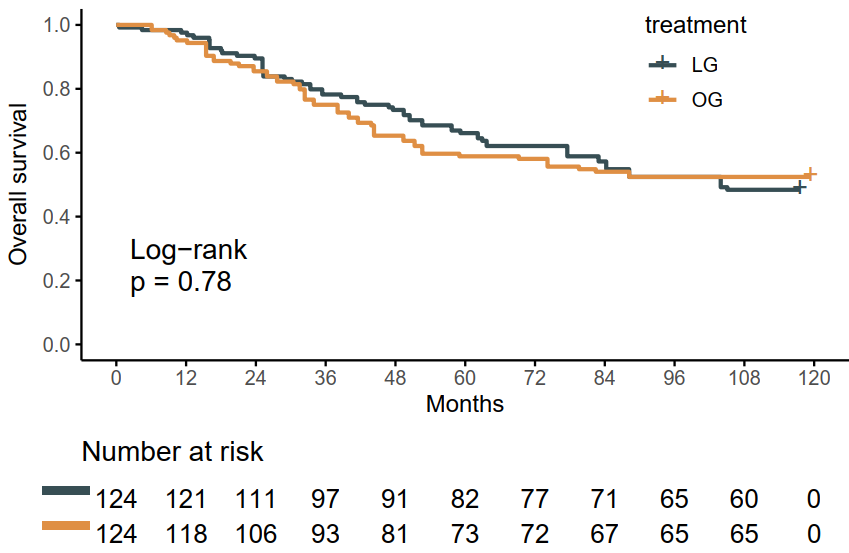 |
| Jeong, 2022  (RFS) | 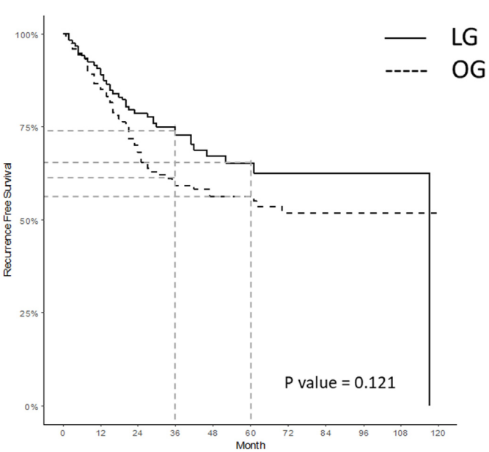 | 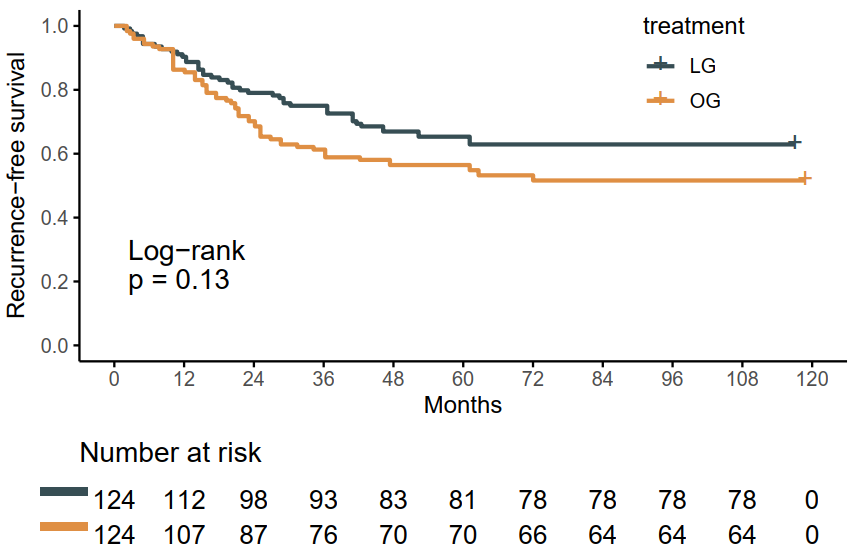 |
| Kuwaraba, 2023  (OS) | 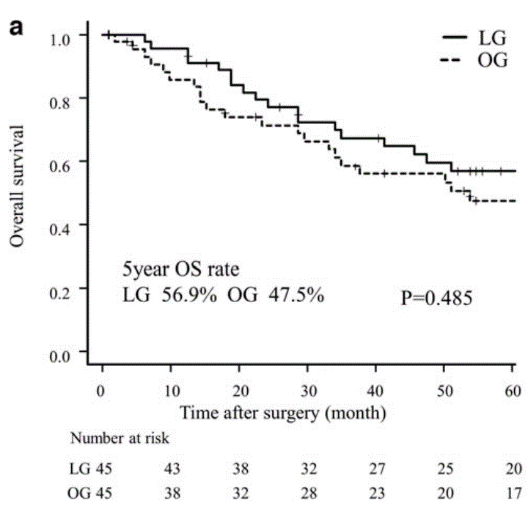 | 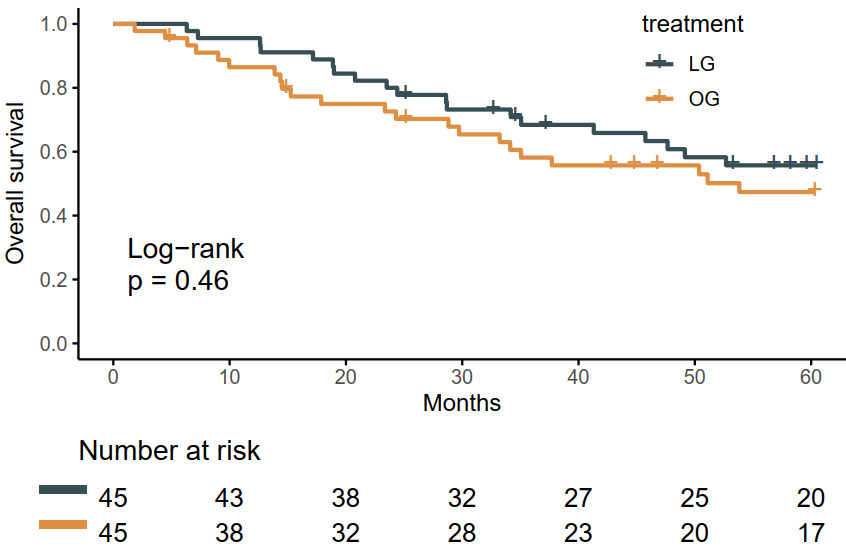 |
| Kuwaraba, 2023  (DFS) | 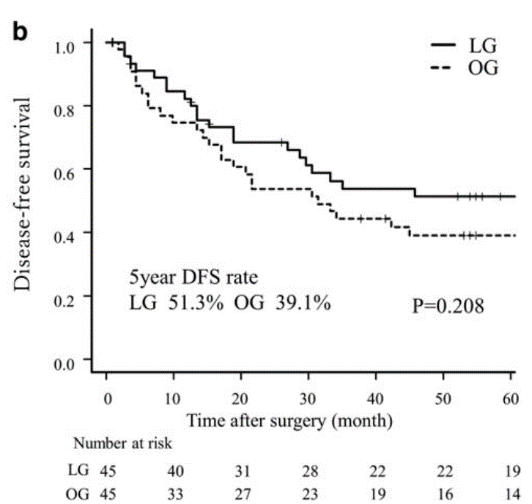 | 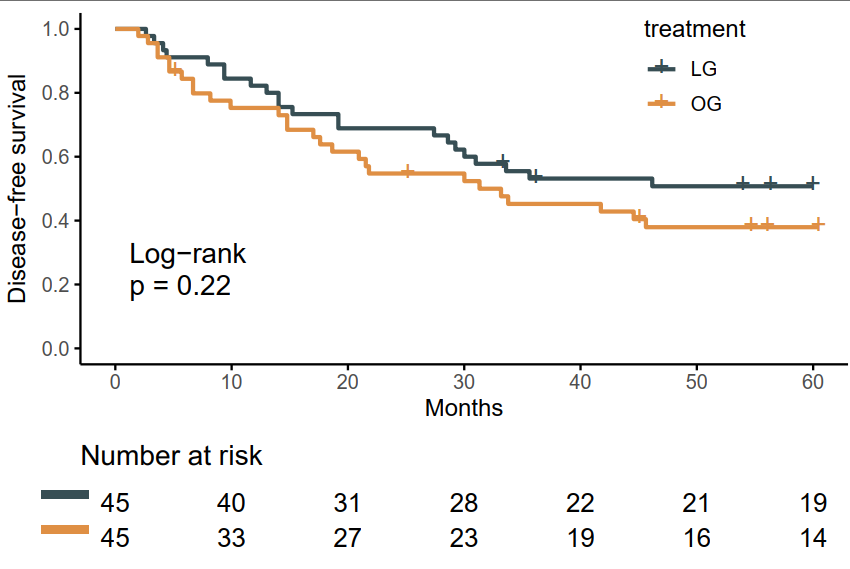 |
| Li, 2019  (OS) | 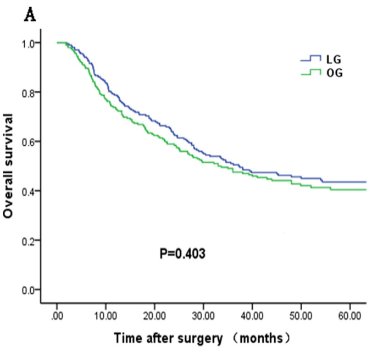 | 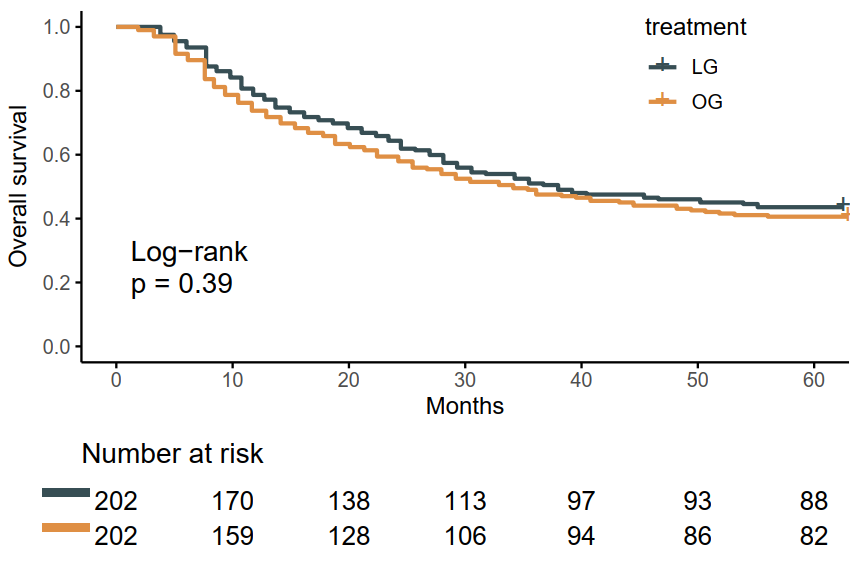 |
| Li, 2019  (DFS) | 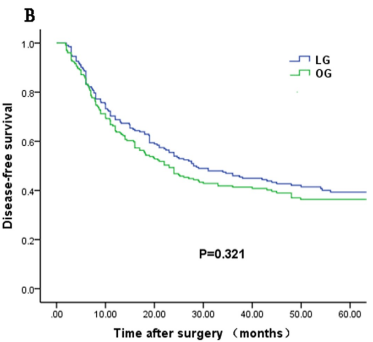 | 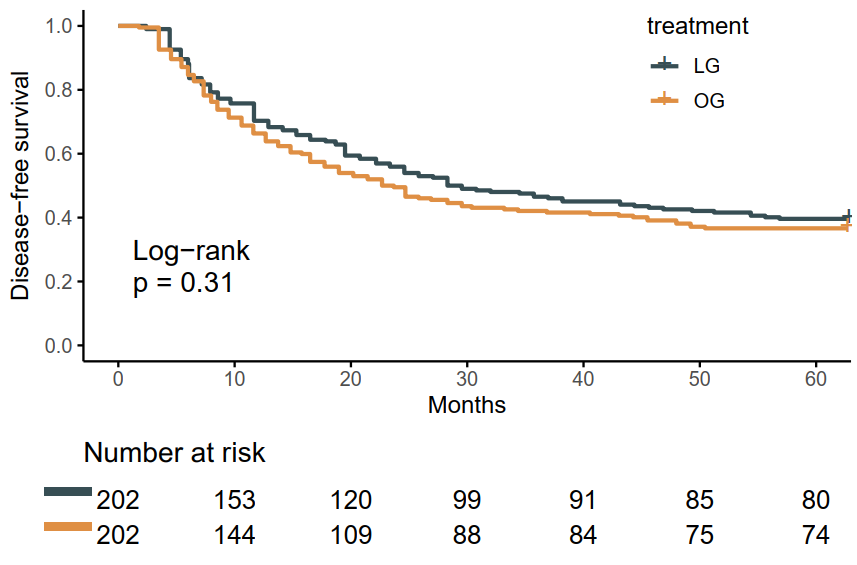 |
| Long, 2021  (OS) | 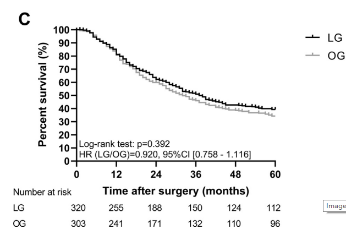 | 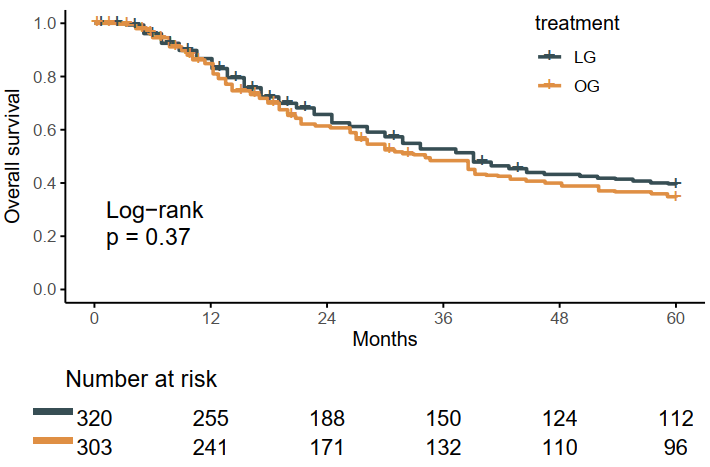 |
| Long, 2021  (DFS) | 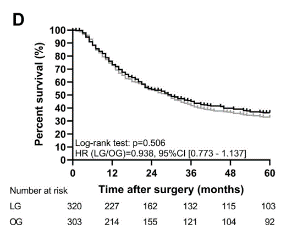 | 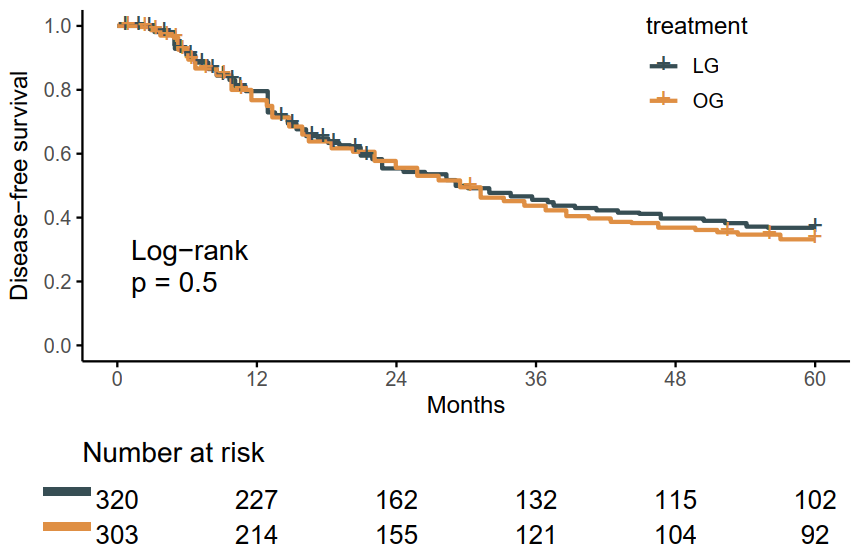 |
| Long, 2022  (OS) | 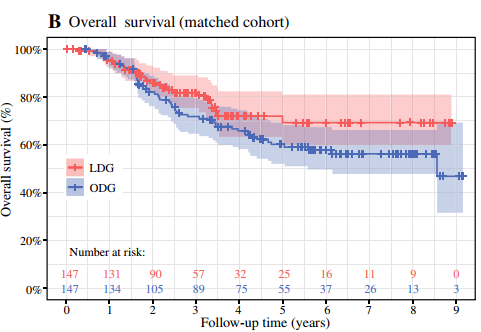 | 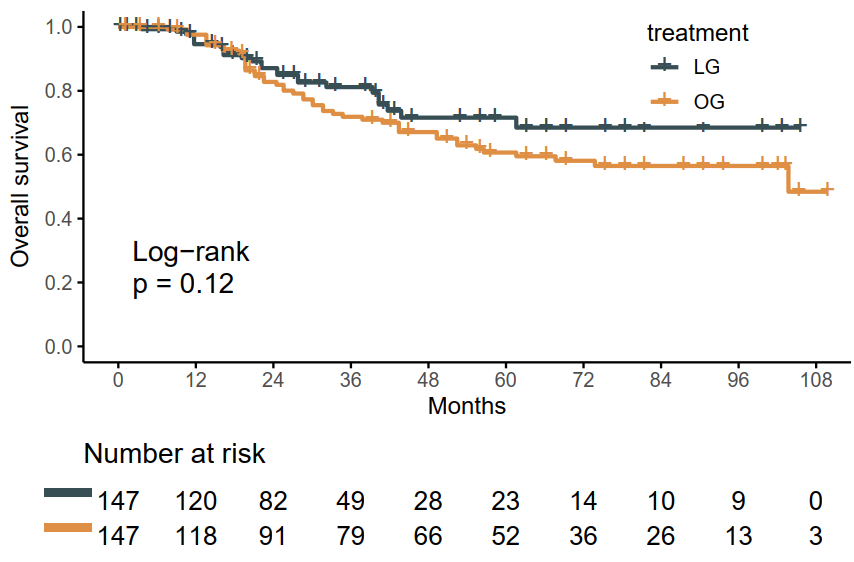 |
| Long, 2022  (DFS) | 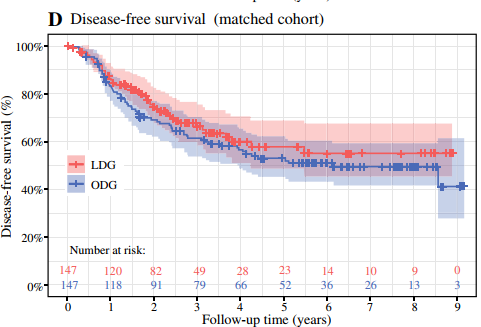 | 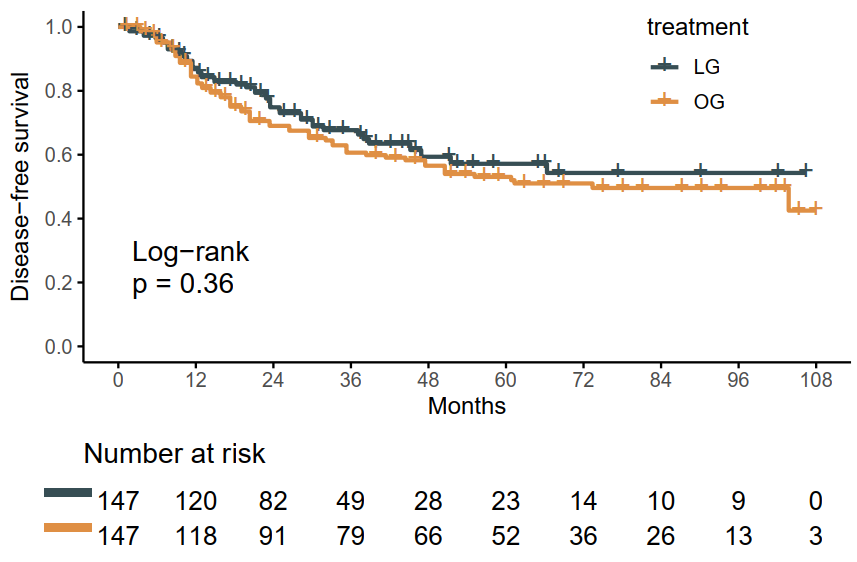 |
| Pang, 2021  (OS) | 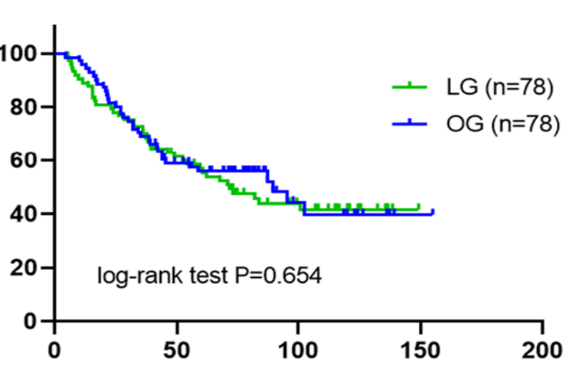 | 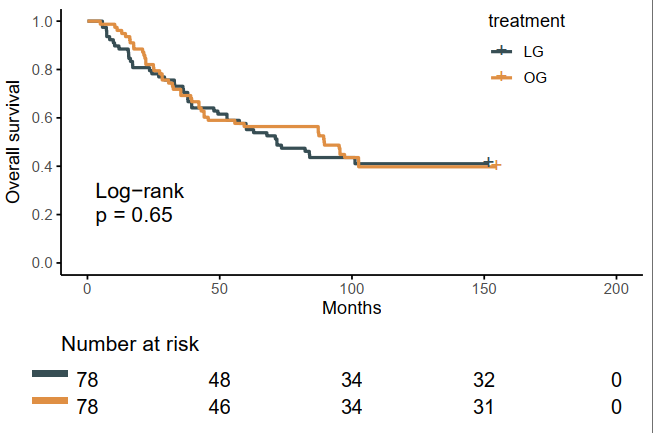 |
