# Supplementary material for: Laparoscopic versus open gastrectomy for nonmetastatic T4a gastric cancer: a meta-analysis of reconstructed individual participant data from propensity score-matched studies
Source: World J Surg Oncol. 2024 May 29;22:143. doi: 10.1186/s12957-024-03422-5 (PMC11134691; doi:10.1186/s12957-024-03422-5)
Supplement: Supplementary file 6 — Supplementary Material 6 [file 12957_2024_3422_MOESM6_ESM.docx]

**A. Overall survival**


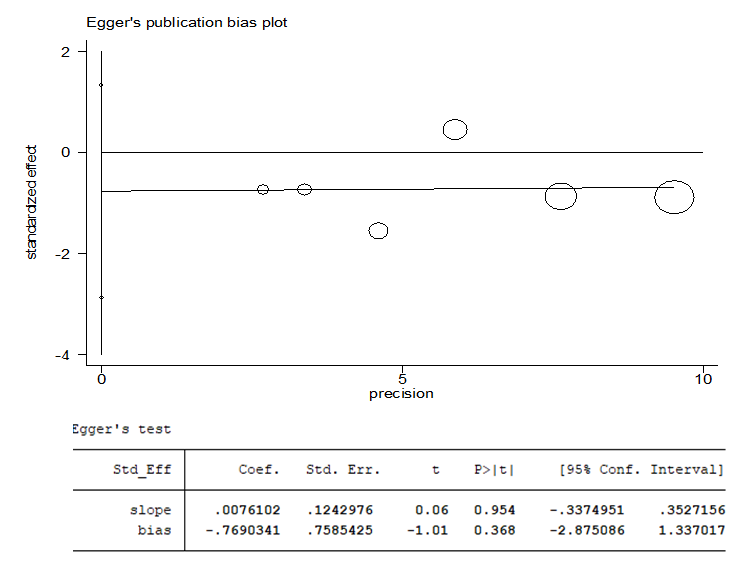


**B. Disease-free survival**


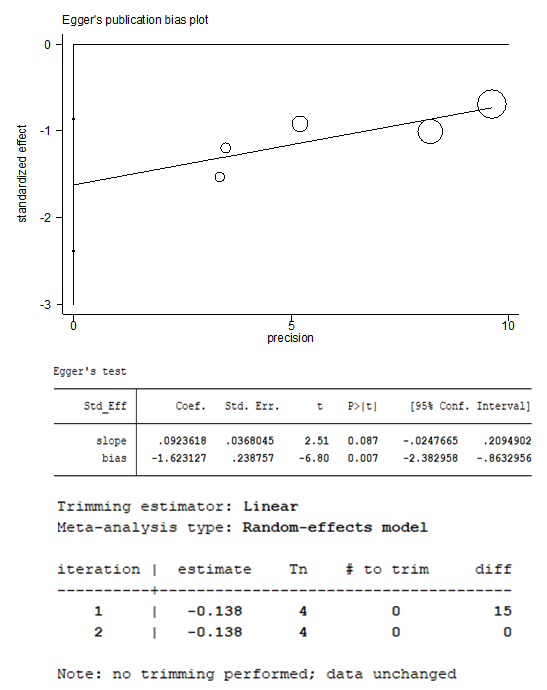


**C. Operative time**

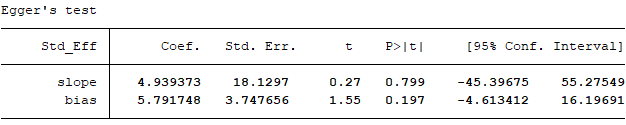


**D. Estimated blood loss**


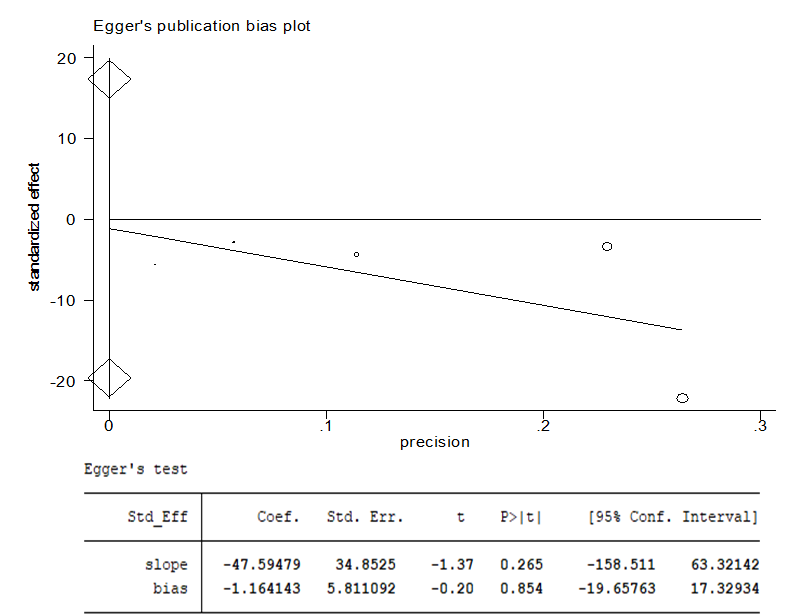


**E. No. of retrieved lymph nodes**


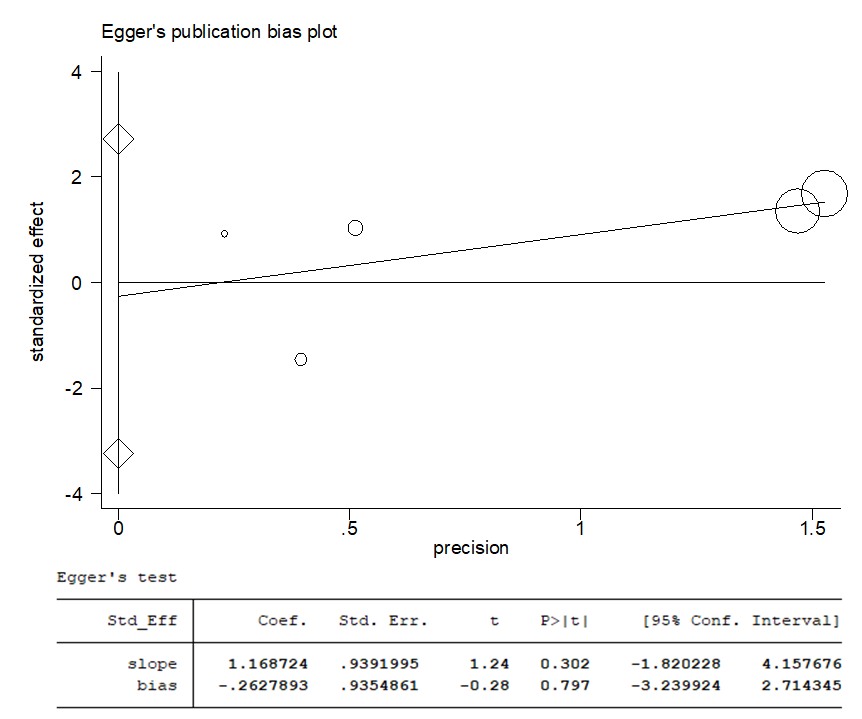


**F. Time to first liquid intake**


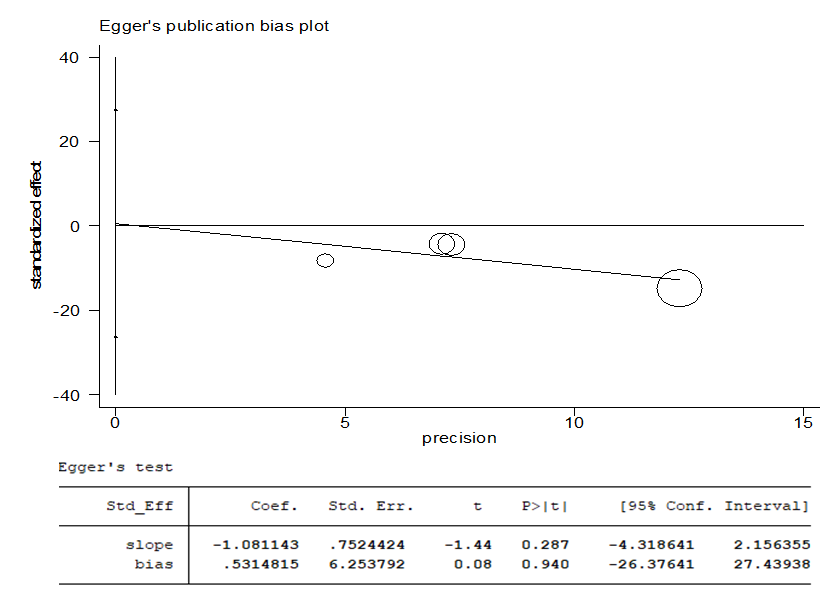


**G. Time to first flatus**


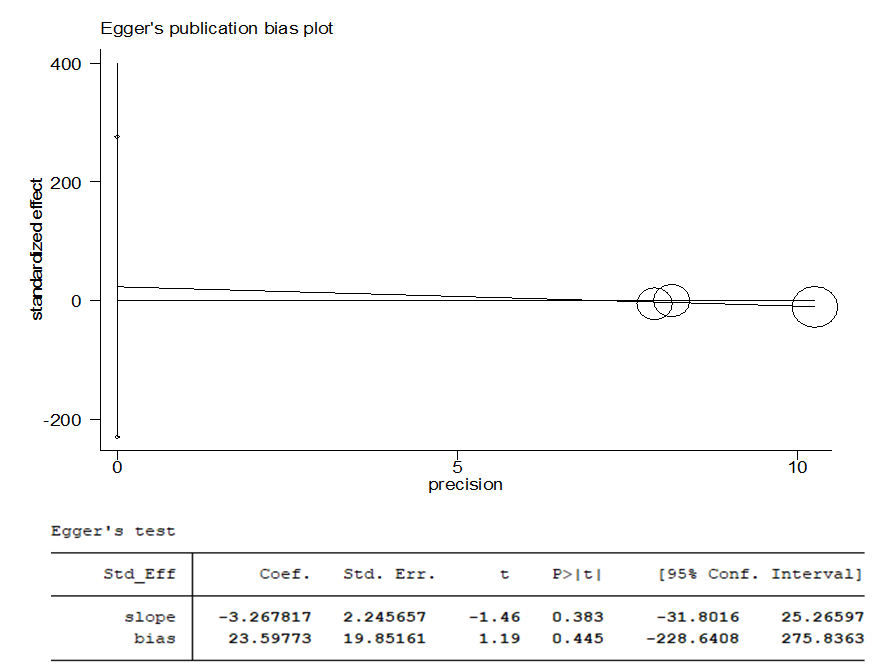


**H. Postoperative hospital stay**


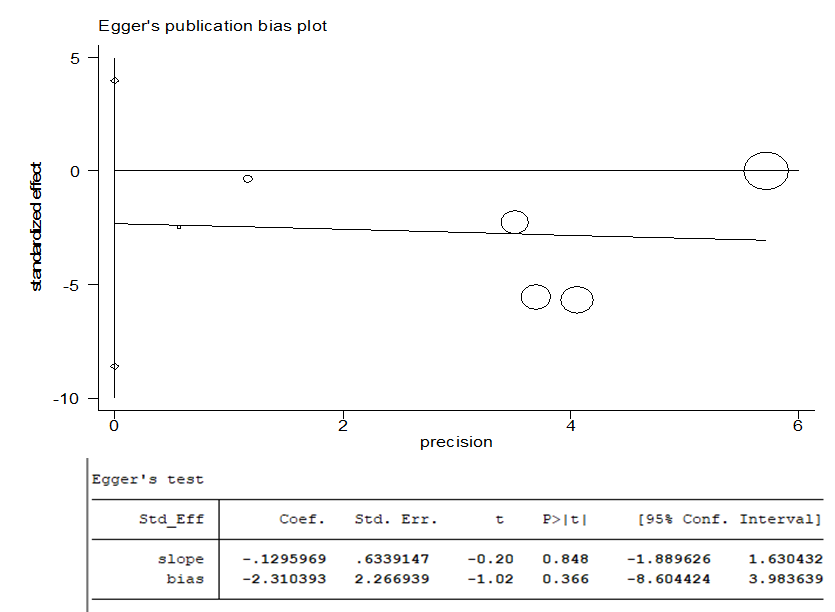


**I. Overall morbidity**


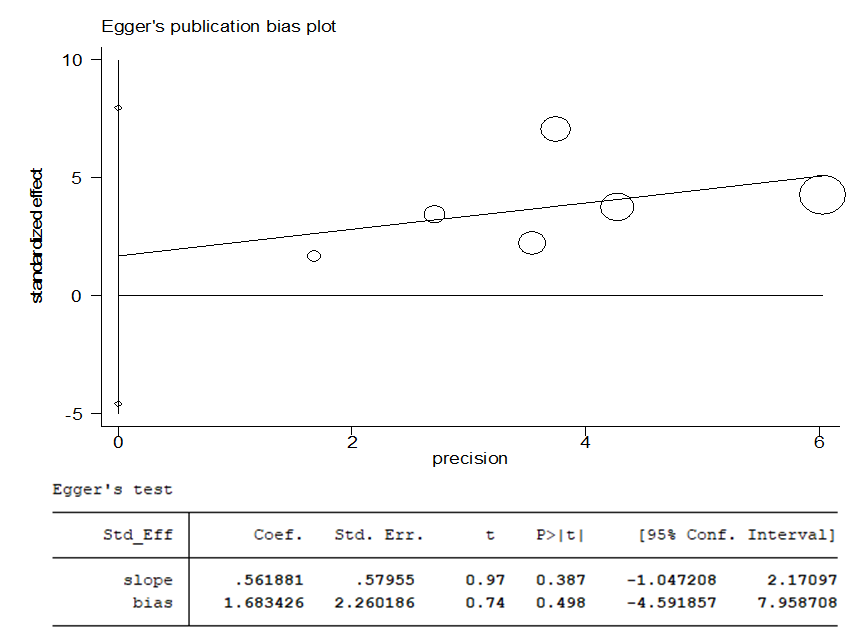


**J. Major morbidity**


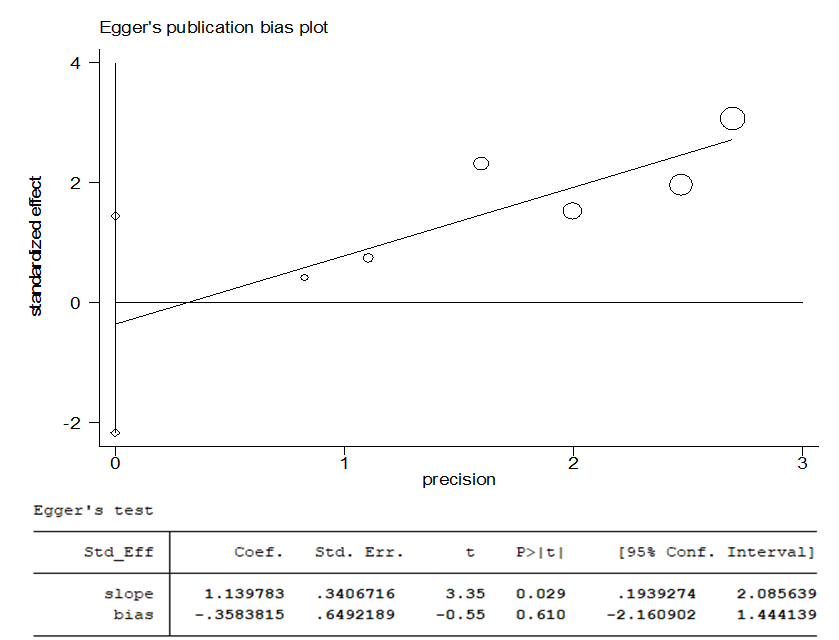


**K. Total recurrence**


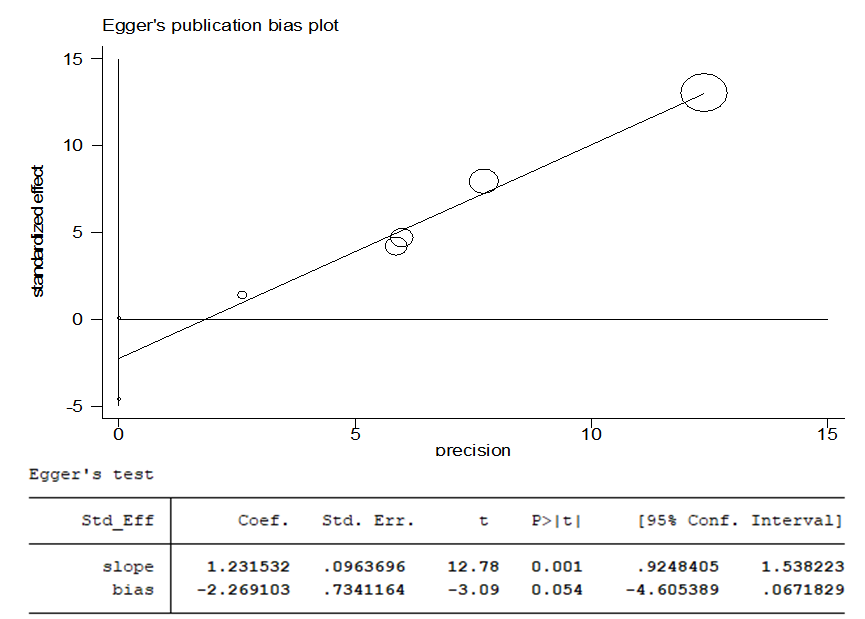


**L. Peritoneal recurrence**


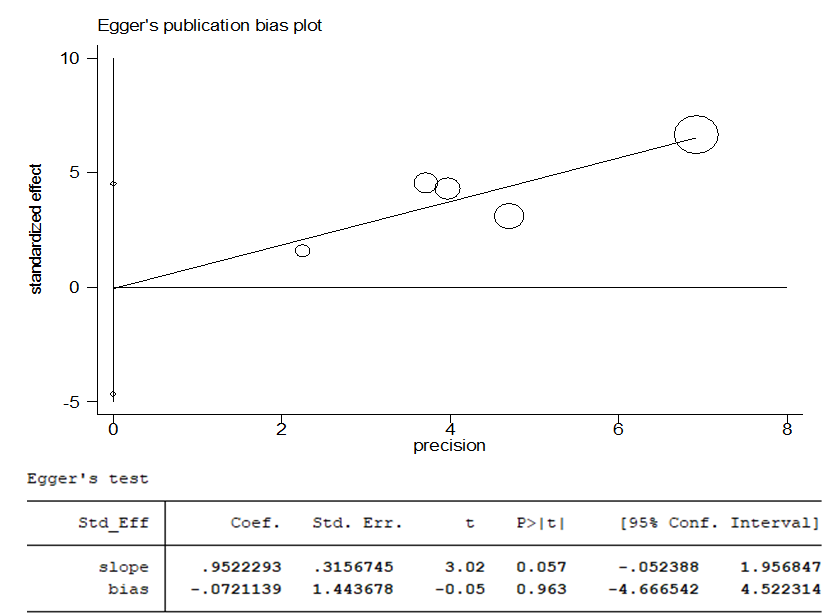


**M. Local recurrence**


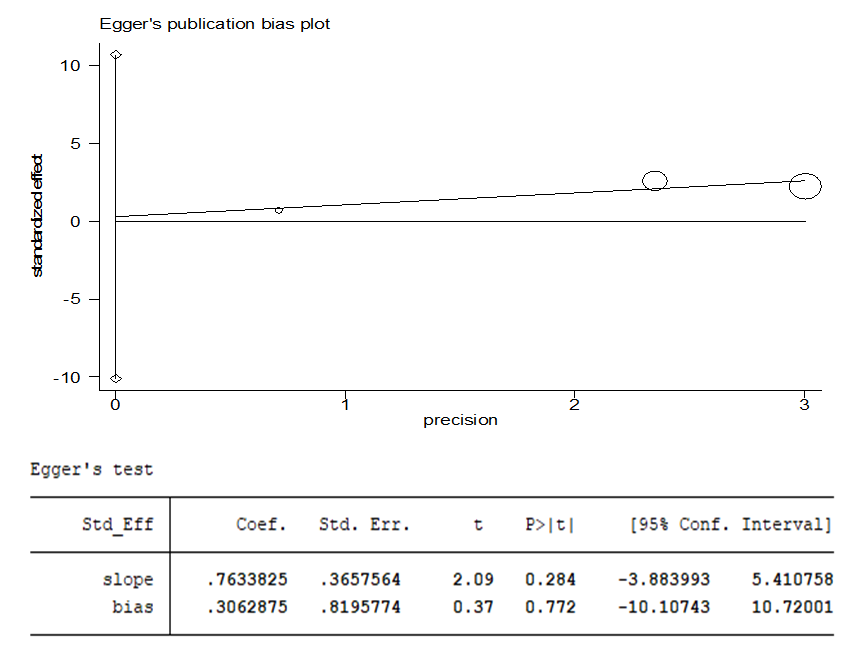


**N. Distant LN recurrence**


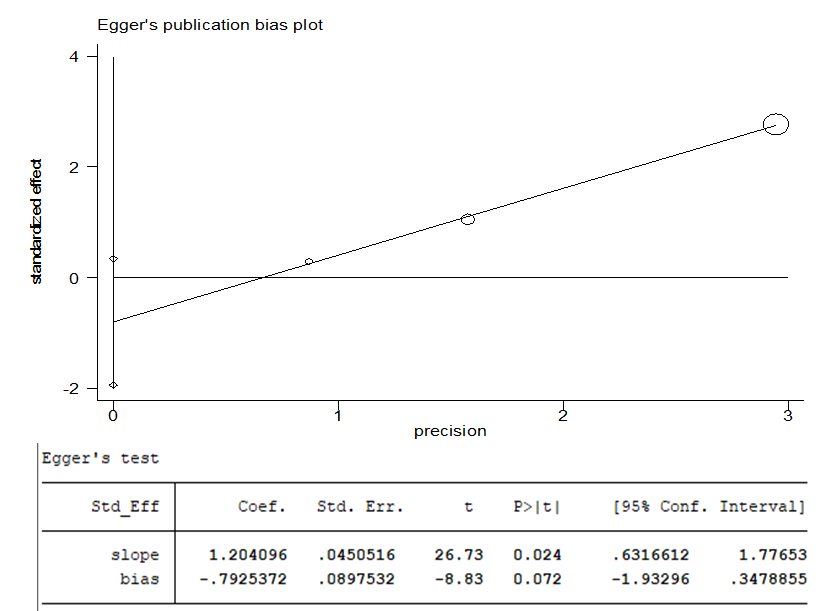


**O. Hematogenous recurrence**


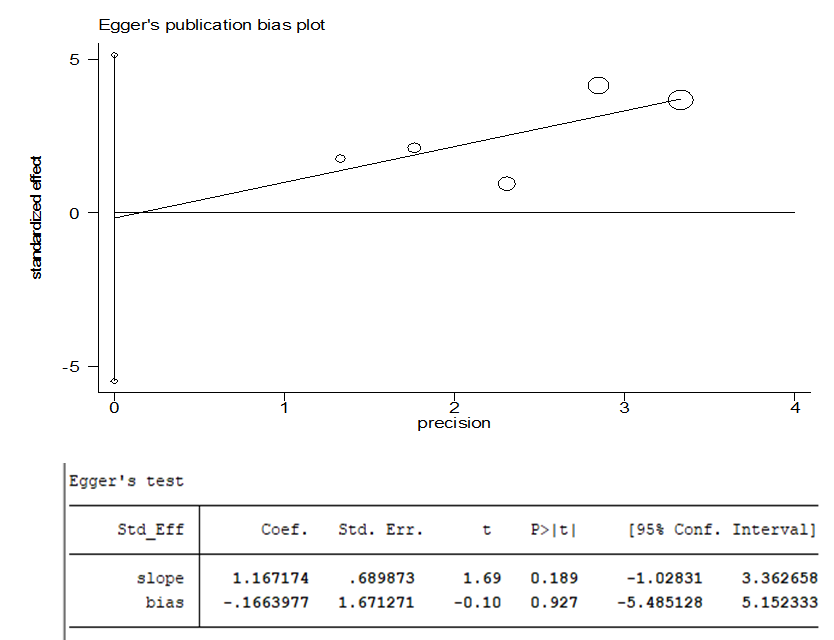


**P. Mixed recurrence**


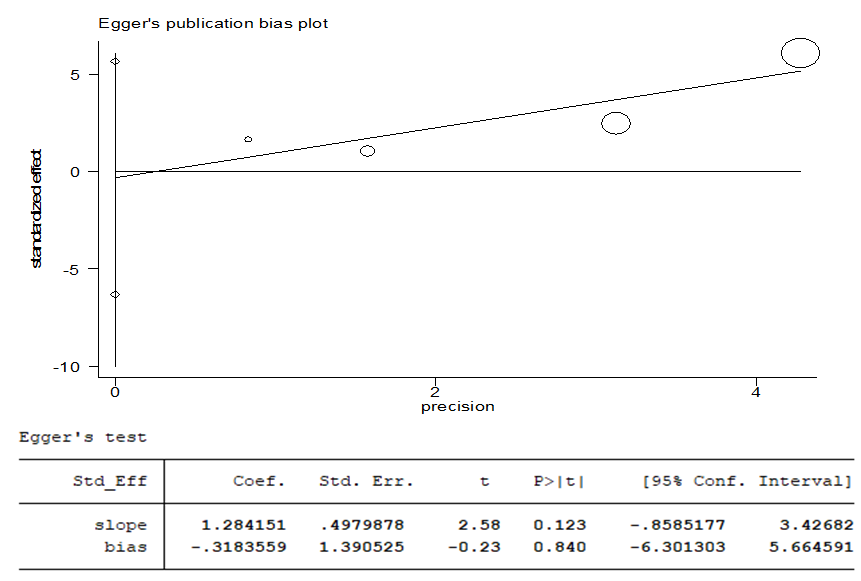


Supplementary file item 7. Egger’s tests assessing the potential publication bias for primary and secondary outcomes. Trim and fill analysis was applied for disease-free survival.
